# Longitudinal Gut Microbiota Changes in Women with Breast Cancer-Related Lymphedema Undergoing a Lifestyle Intervention

**DOI:** 10.3390/ijms27209008

**Published:** 2026-10-11

**Authors:** Yolanda Ruiz-Molina, Marina Padial, Cristina Rodríguez-Díaz, Sheila González-Salvatierra, María del Mar Martín-Bravo, Isaac Plaza-Andrades, Lucía Aránega-Martín, Francisca Rodríguez-Pacheco, Alejandro Chicharro-Parras, Corina Verónica Sasso, María Isabel Queipo-Ortuño, Gabriel Olveira

**Affiliations:** 1 Servicio de Medicina Física y Rehabilitación, Hospital Universitario Virgen de la Victoria, 29010 Malaga, Spain; 2 Department of Medicine and Dermatology, University of Málaga, 29010 Malaga, Spain; 3 Servicio de Endocrinología y Nutrición, Hospital Regional Universitario de Málaga, 29010 Malaga, Spain; 4 IBIMA Plataforma BIONAND, Instituto de Investigación Biomédica de Málaga, 29010 Malaga, Spain; 5 Unidad de Gestión Clínica de Aparato Digestivo, Hospital Universitario Virgen de la Victoria, 29010 Malaga, Spain; 6 Instituto de Salud Carlos III, CIBER of Frailty and Healthy Aging (CIBERFES), 28029 Madrid, Spain; 7 Servicio de Medicina Física y Rehabilitación, Hospital Regional Universitario de Málaga, 29010 Malaga, Spain; 8 Unidad de Gestión Clínica de Oncología Médica, Hospital Universitario Virgen de la Victoria, 29010 Malaga, Spain; 9 Department of Surgical Specialties, Biochemistry and Immunology, University of Málaga, 29010 Malaga, Spain; 10 Instituto de Salud Carlos III, CIBER of Diabetes and Associated Metabolic Diseases (CIBERDEM), 28029 Madrid, Spain

**Keywords:** *Bifidobacterium*, breast cancer-related lymphedema, exercise, gut microbiota, mediterranean diet, obesity

## Abstract

Breast cancer-related lymphedema (BCRL) is closely associated with obesity, yet the gut microbiota remains poorly characterized in this population. We longitudinally evaluated gut microbiota profiles in 48 women with overweight or obesity and BCRL who were randomized to either a multimodal lifestyle intervention combining an energy-restricted Mediterranean-style diet and exercise (*n* = 28) or control care (*n* = 20). Fecal samples collected at baseline, 3 months, and 6 months (144 samples) underwent 16S rRNA gene sequencing. The intervention resulted in greater reductions in body weight, body mass index, and fat mass over time (group × time *p* < 0.001, *p* = 0.001, and *p* = 0.002, respectively). No significant group × time interaction was observed for alpha diversity or overall microbial community composition based on Bray–Curtis dissimilarity (PERMANOVA *R*^2^ = 0.006, *F* = 0.459, *p* = 0.324). Exploratory DESeq2 differential abundance analyses identified FDR-significant taxon-specific differences, including higher abundances of Barnesiellaceae and Desulfovibrionaceae in the intervention group and of *Prevotella_7* and *Lachnospiraceae_UCG_010* in controls (FDR-adjusted *p* < 0.05). These DESeq2 findings were not confirmed in mixed-effects sensitivity analyses after FDR correction. *Prevotella_7* was more abundant in controls at baseline and at 6 months. Participant-blocked group × time models, which were given inferential priority, yielded FDR-significant baseline-to-3-month interaction tests for Izemoplasmatales and *Frisingicoccus,* although the corresponding shrunken effect estimates were close to zero. Secondary, hypothesis-generating analyses based on supplementation status, supplement adherence, and clinically relevant weight-loss response identified additional taxon-specific patterns associated with supplementation and adherence, including a tentative species-level association involving *Bifidobacterium bifidum* among supplemented participants, whereas no significant microbiota differences were observed by weight-loss response. Overall, these findings provide no evidence of broad restructuring of the gut microbial community but identify exploratory taxon-specific signals that warrant confirmation in larger longitudinal studies using strain-resolved and functional approaches.

## 1. Introduction

Breast cancer-related lymphedema (BCRL) is a chronic complication of breast cancer treatment resulting from impaired lymphatic drainage and progressive tissue remodeling [1]. Beyond interstitial fluid accumulation, BCRL is characterized by persistent inflammation, adipose tissue deposition, and fibrosis, which contribute to sustained limb swelling, functional impairment, and reduced quality of life among breast cancer survivors [2,3,4]. Excess adiposity is particularly relevant in this setting, as overweight and obesity are associated with an increased risk of BCRL and may further compromise lymphatic function through adipose tissue expansion, chronic low-grade inflammation, and impaired lymphatic transport [5]. Conversely, chronic lymphatic dysfunction may itself promote local adipose accumulation and inflammatory tissue remodeling, supporting a bidirectional relationship between lymphatic dysfunction and adiposity [6]. This biological interplay provides a rationale for targeting modifiable metabolic and lifestyle-related factors in women with BCRL and excess adiposity.

The gut microbiota has emerged as an important interface between diet, adipose tissue, metabolism, and immune regulation. Microbial communities and their metabolites influence energy homeostasis, intestinal barrier integrity, and local and systemic immune responses through pathways involving short-chain fatty acids, bile acids, lipopolysaccharides, and other microbially derived signals [7,8]. Alterations in gut microbial composition and function have been associated with obesity and obesity-related metabolic dysfunction, although substantial interindividual and methodological heterogeneity remains across studies [9]. Given the contribution of gut microbial alterations to obesity-associated metabolic and inflammatory disturbances [10] and the close interplay between excess adiposity, inflammation, and lymphatic dysfunction, the gut microbiota could plausibly contribute to the metabolic and inflammatory environment in which BCRL develops or persists. However, to our knowledge, no previous study has directly characterized the gut microbiota using 16S rRNA gene sequencing in women with BCRL, and the potential role of the gut microbiota in the biological response to lifestyle-based interventions in this population remains uncharacterized. A recent pilot study in women with lipedema, a distinct condition that also involves adipose tissue and lymphatic alterations, characterized gut microbiota profiles using 16S rRNA gene sequencing [11], providing a relevant clinical analogue but not direct evidence for BCRL.

Importantly, the gut microbiota is responsive to modifiable lifestyle exposures, providing a potential mechanistic link between lifestyle interventions and metabolic health. Mediterranean-style dietary patterns have been associated with changes in gut microbial composition, metabolome, and microbiota-derived metabolites [12,13], including in the context of an energy-reduced intervention combined with physical activity promotion [13]. Physical activity may also influence gut microbial diversity and composition [14], although reported effects are heterogeneous, particularly among individuals with overweight or obesity [15]. Beyond these lifestyle factors, nutritional strategies incorporating microbiota-targeted components may provide a more direct approach to modulating the gut microbial ecosystem. In particular, *Bifidobacterium animalis* subsp. *lactis* CECT 8145 (BPL1) has been evaluated in adults with abdominal obesity, with reported effects on adiposity-related outcomes and gut microbiota-related parameters [16]. Together, these observations support the investigation of gut microbiota dynamics within multimodal lifestyle interventions targeting obesity-related conditions.

Accordingly, the present study was conducted as an exploratory gut microbiota substudy of a previously reported randomized lifestyle intervention in women with overweight or obesity and BCRL [17]. The aim was to characterize longitudinal patterns in gut microbial diversity and taxonomic composition and to explore whether these patterns differed over time by intervention group, supplementation status, supplement adherence, and clinically relevant weight-loss response.

## 2. Results

### 2.1. Characteristics of the Study Population

Table 1 summarizes the baseline characteristics of the overall study population and compares participants by study group. Overall, the intervention and control groups were broadly comparable at baseline. The only significant between-group difference was observed in the prevalence of dyslipidemia, which was higher in the control group than in the intervention group (*p* = 0.007). No significant differences were observed in the other demographic, lifestyle, cancer-related, comorbidity, anthropometric, body composition, lymphedema-related, dietary, or biochemical variables, including fat-free mass, affected limb volume, body mass index (BMI), phase angle, and absolute and relative interlimb volume differences.

### 2.2. Changes in Clinical Outcomes

Changes in clinical outcomes during follow-up are summarized in Table 2. Compared with the control group, participants assigned to the intervention group showed greater reductions in body weight at both 3 months (*p* = 0.022) and 6 months (*p* = 0.002), with a significant group × time interaction (*p* < 0.001). Similar findings were observed for BMI, with greater reductions in the intervention group at 3 months (*p* = 0.044) and 6 months (*p* = 0.002), together with a significant group × time interaction (*p* = 0.001). Fat mass also decreased to a greater extent in the intervention group at both 3 months (*p* = 0.004) and 6 months (*p* = 0.008), with a significant group × time interaction (*p* = 0.002). No significant between-group differences or group × time interactions were observed for waist circumference, fat-free mass, or phase angle.

No significant between-group differences or group × time interactions were observed for any lymphedema-related outcome, including affected limb volume, absolute interlimb volume difference, and relative interlimb volume difference.

Regarding dietary outcomes, energy intake decreased to a greater extent in the intervention group at 3 months (*p* = 0.045), whereas the between-group difference at 6 months did not reach statistical significance (*p* = 0.080). A significant group × time interaction was observed for energy intake (*p* = 0.040). Changes in Mediterranean diet adherence were comparable between groups at both follow-up time points, with no significant group × time interaction (*p* = 0.133).

### 2.3. Taxonomic Composition and Diversity of the Gut Microbiota

Following quality filtering of the 16S rRNA gene sequencing data, samples yielding fewer than 3000 reads were excluded, and the remaining samples were rarefied to an even sequencing depth of 3000 reads per sample for diversity analyses. Taxonomic assignment identified 255 bacterial taxa at the genus level or, when genus-level assignment was not available, at the nearest resolved taxonomic level, with a mean Good’s coverage of 99%, indicating high sample coverage at the selected rarefaction depth.

Alpha diversity was assessed using the Chao1 richness, Shannon, Simpson, and inverse Simpson indices. Longitudinal mixed-effects models including participant as a random intercept showed no significant group × time interaction for any alpha-diversity index (all *p* ≥ 0.430; Holm-adjusted *p* = 1.000 for all indices). Consistently, no significant differences were observed between the intervention and control groups at baseline (all *p* ≥ 0.198), 3 months (all *p* ≥ 0.746), or 6 months (all *p* ≥ 0.363). Likewise, changes from baseline to 3 months (all *p* ≥ 0.427) and from baseline to 6 months (all *p* ≥ 0.714) did not differ significantly between groups (Supplementary Table S1).

**Figure 1:**
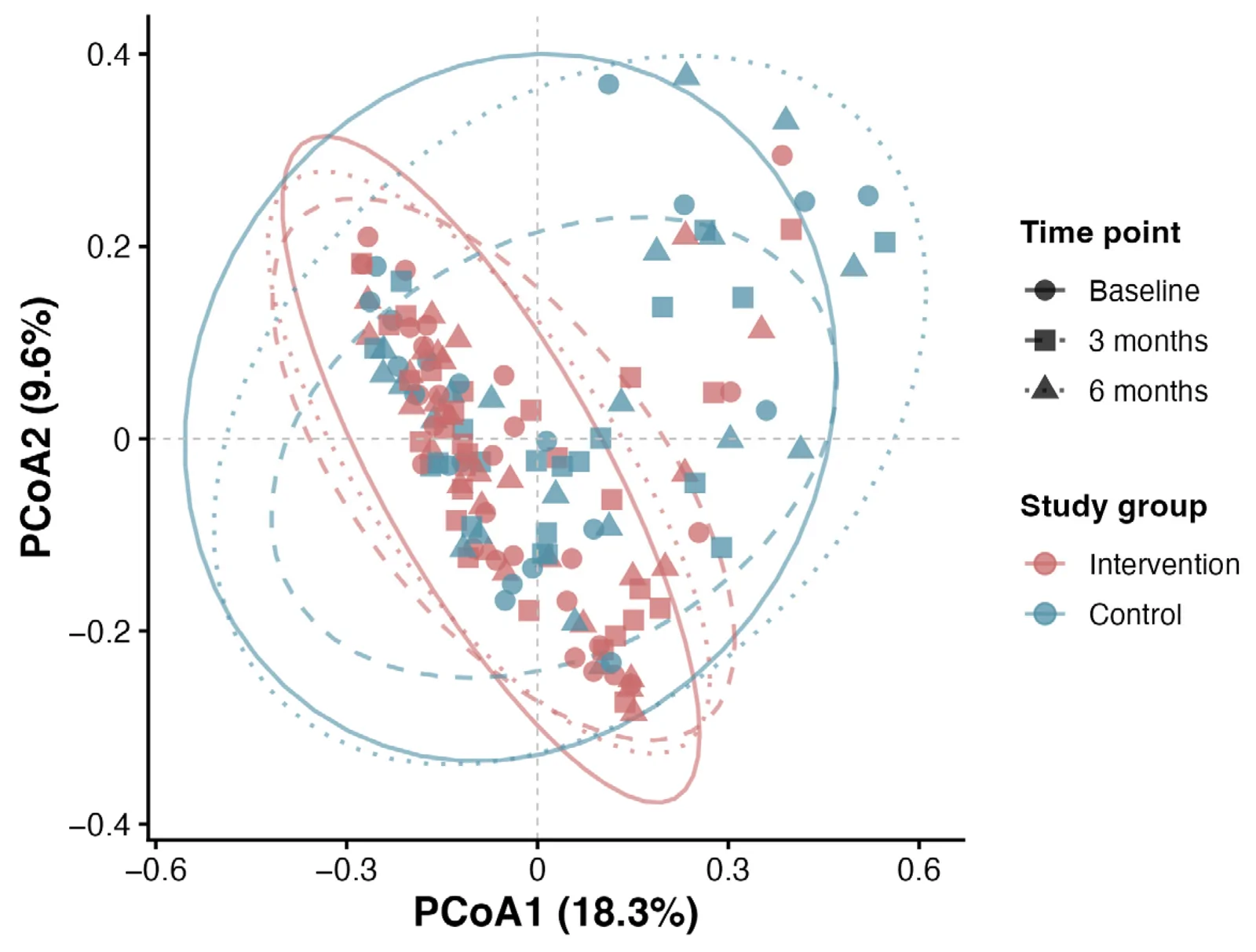
Bray–Curtis beta diversity according to study group and follow-up time. Principal coordinates analysis (PCoA) based on Bray–Curtis dissimilarity showing gut microbial community composition in the intervention and control groups at baseline, 3 months, and 6 months. Points represent individual samples, and ellipses indicate clustering according to group–time combinations. PERMANOVA with permutations restricted by participant showed no significant group × time effect on microbial community composition (*R*^2^ = 0.006, *F* = 0.459, *p* = 0.324).

Beta diversity was evaluated using Bray–Curtis dissimilarity across study groups and sampling time points. Principal coordinates analysis showed substantial overlap among group–time combinations, with no clear separation between intervention and control samples across follow-up. PERMANOVA, with permutations restricted by participant to account for repeated measurements, did not detect a significant group × time effect on microbial community composition (*R*^2^ = 0.006, *F* = 0.459, *p* = 0.324). Multivariate dispersion did not differ significantly among group–time combinations (PERMDISP, *p* = 0.223), indicating comparable dispersion across groups and time points (Figure 1).

### 2.4. Differentially Abundant Taxa According to Study Group

Exploratory taxonomic analyses identified several group-specific patterns across follow-up (Supplementary Figure S1), despite the absence of differences in global diversity measures. 

**Figure 2:**
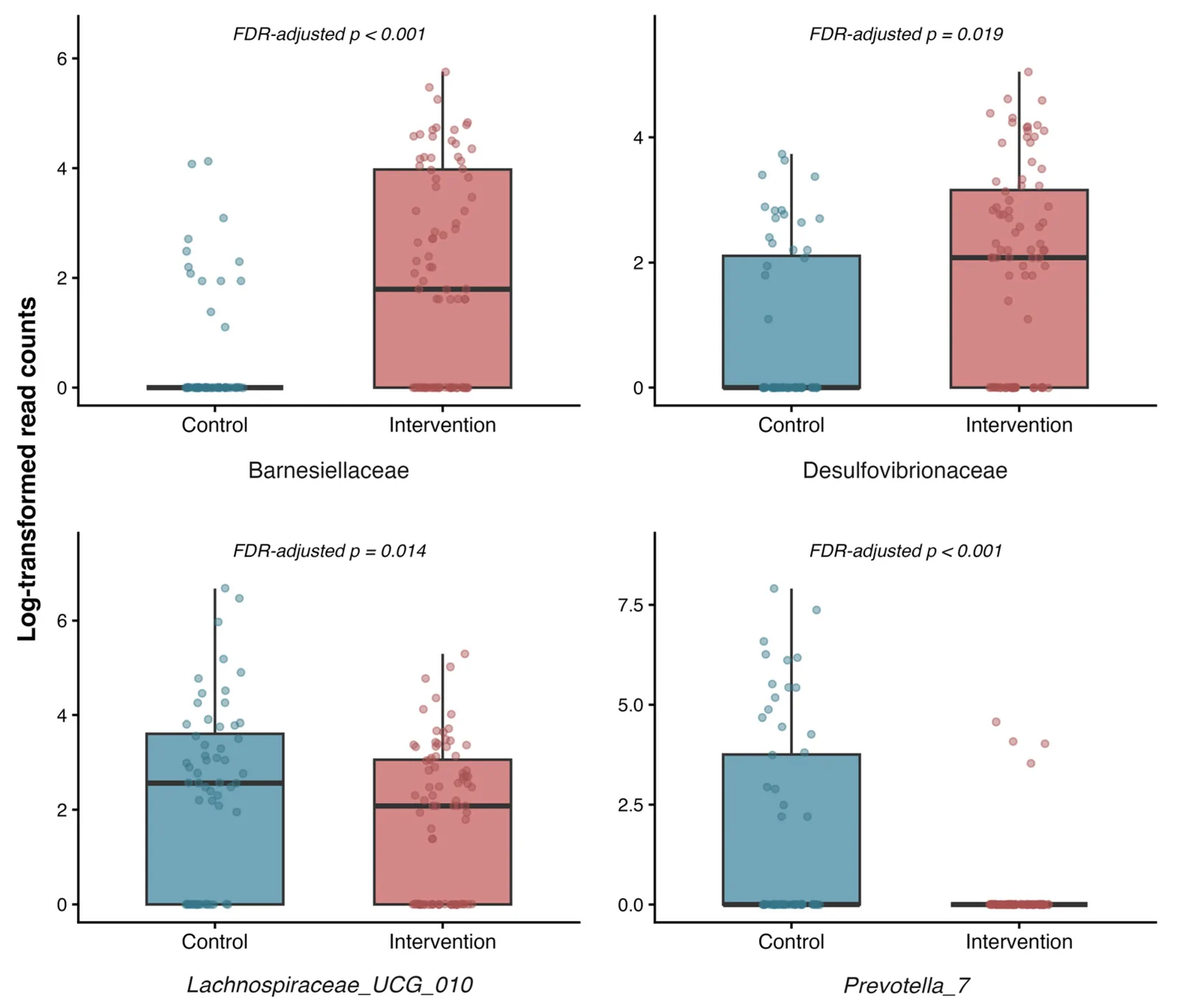
Overall differentially abundant bacterial taxa between the intervention and control groups across follow-up. Boxplots show the distribution of log-transformed read counts for bacterial taxa identified as significantly different between the intervention and control groups in the overall DESeq2 analysis including study group and sampling time. Boxes represent the interquartile range, horizontal lines indicate the median, whiskers extend to 1.5 times the interquartile range, and points represent individual samples. *p*-values were adjusted for multiple comparisons using the Benjamini–Hochberg FDR method, with an FDR-adjusted *p* < 0.05 considered statistically significant. FDR-adjusted *p*-values are displayed within each panel.

An overall exploratory DESeq2 analysis across follow-up, adjusted for sampling time, identified four taxa that differed significantly between the intervention and control groups after false discovery rate (FDR) correction. Barnesiellaceae (shrunken log2FoldChange = 2.248, FDR-adjusted *p* < 0.001) and Desulfovibrionaceae (shrunken log2FoldChange = 1.443, FDR-adjusted *p* = 0.019) were enriched in the intervention group, whereas *Prevotella_7* (shrunken log2FoldChange = −1.946, FDR-adjusted *p* < 0.001) and *Lachnospiraceae_UCG_010* (shrunken log2FoldChange = −1.419, FDR-adjusted *p* = 0.014) were enriched in the control group (Figure 2; Supplementary Table S3A). The corresponding prevalence and zero-count frequencies are reported in Supplementary Table S3B to provide additional context for taxa with sparse distributions. Mixed-effects modeling accounting for repeated measurements was performed as a sensitivity analysis; the direction of the four associations was preserved, but none remained significant after FDR correction. Accordingly, these DESeq2 findings should be interpreted as exploratory taxon-specific signals rather than robust longitudinal evidence.

**Figure 3:**
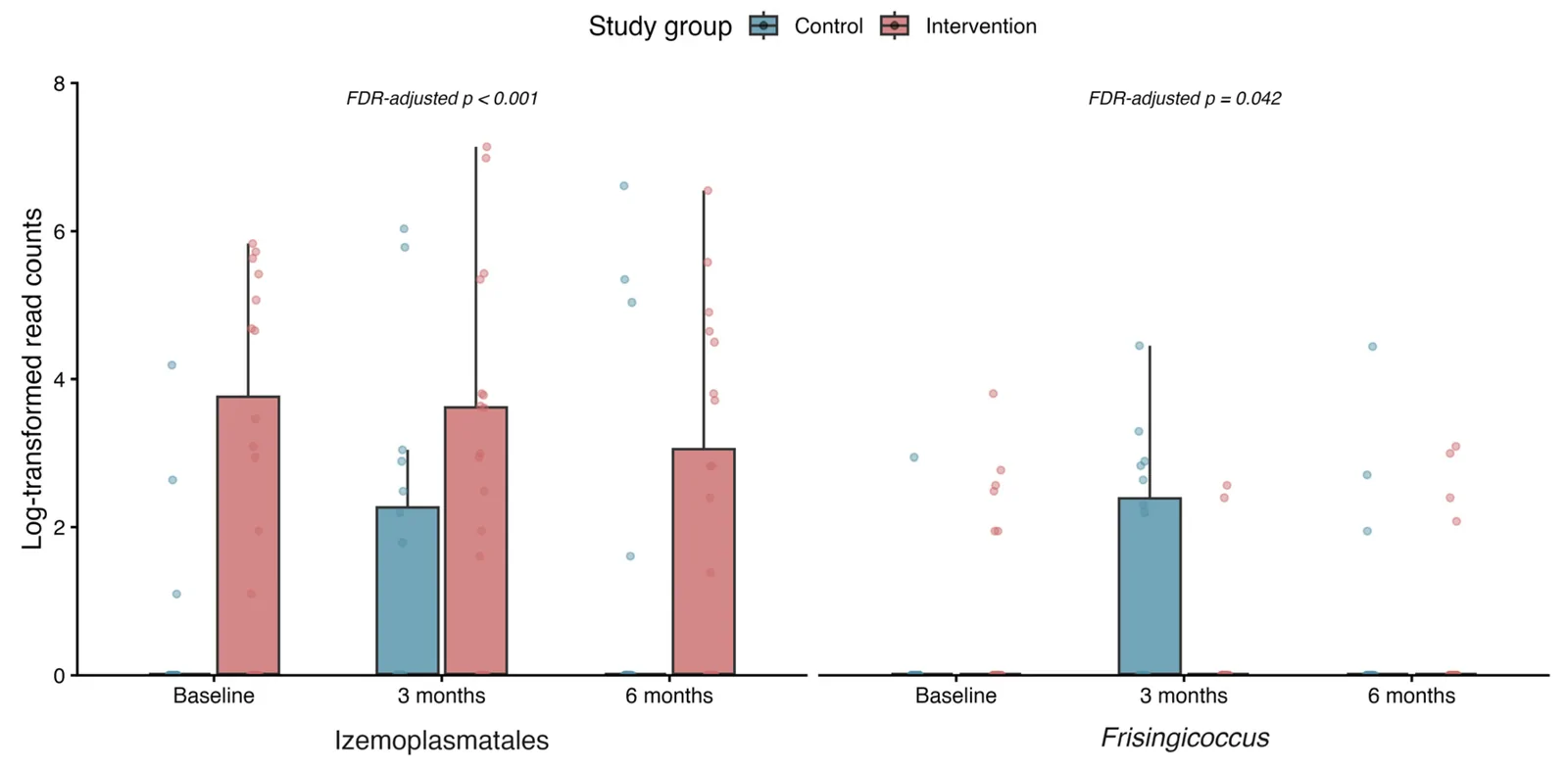
Longitudinal trajectories of bacterial taxa showing FDR-significant group × time interactions between the intervention and control groups. Boxplots show the distribution of log-transformed read counts for Izemoplasmatales and *Frisingicoccus* at baseline, 3 months, and 6 months according to study group. Boxes represent the interquartile range, horizontal lines indicate the median, whiskers extend to 1.5 times the interquartile range, and points represent individual samples. FDR-adjusted *p*-values for the group × time interaction from baseline to 3 months are displayed in each panel. Effect-size estimates reported in the text were stabilized using DESeq2 *lfcShrink*, and prevalence and zero-count frequencies are provided in Supplementary Table S3B.

Participant-blocked longitudinal analyses additionally identified FDR-significant group × time interaction tests for changes from baseline to 3 months for Izemoplasmatales (shrunken interaction log2FoldChange = 0.042, FDR-adjusted *p* < 0.001) and *Frisingicoccus* (shrunken interaction log2FoldChange = −0.127, FDR-adjusted *p* = 0.042) (Figure 3). Both interaction coefficients were substantially attenuated after log2 fold-change shrinkage. No taxa showed significant differential changes between groups from baseline to 6 months after FDR correction.

Time-stratified analyses identified a significant baseline difference for *Prevotella_7*, which was enriched in the control group (shrunken log2FoldChange = −2.516, FDR-adjusted *p* < 0.001). No taxa remained significantly different between groups at 3 months after FDR correction. At 6 months, *Prevotella_7* was again enriched in the control group (shrunken log2FoldChange = −2.079, FDR-adjusted *p* < 0.001), while *Escherichia-Shigella* was also enriched in controls (shrunken log2FoldChange = −0.169, FDR-adjusted *p* = 0.011) (Supplementary Figure S2).

As a sensitivity analysis of the main intervention-versus-control comparisons, a more stringent ≥20% prevalence threshold retained 94 of the 255 taxa. In the overall time-adjusted analysis, Barnesiellaceae, Desulfovibrionaceae, and *Lachnospiraceae*_UCG_010 remained FDR-significant, whereas *Prevotella_7* was excluded because its prevalence was below 20%. In the participant-blocked longitudinal analysis, the baseline-to-3-month group × time interaction for Izemoplasmatales remained FDR-significant, whereas *Frisingicoccus* was excluded because its overall prevalence was 15.97%. In the time-stratified analyses, *Prevotella_7* was excluded under the ≥20% threshold at both baseline and 6 months because its prevalence at these time points was below 20%. No taxa reached FDR significance at baseline or 3 months, whereas *Escherichia-Shigella* remained FDR-significant at 6 months and was more abundant in controls (shrunken log2FoldChange = −0.044, FDR-adjusted *p* = 0.016). No FDR-significant baseline-to-6-month interactions were identified under the ≥20% threshold.

In a post hoc sensitivity analysis restricted to the 47 participants with available dyslipidemia data, all four taxa identified in the overall time-adjusted analysis retained the same direction of association and remained FDR-significant after additional adjustment for baseline dyslipidemia: Barnesiellaceae (shrunken log2FoldChange = 2.74, FDR-adjusted *p* < 0.001), Desulfovibrionaceae (shrunken log2FoldChange = 2.15, FDR-adjusted *p* < 0.001), *Lachnospiraceae*_UCG_010 (shrunken log2FoldChange = −1.80, FDR-adjusted *p* = 0.0045), and *Prevotella_7* (shrunken log2FoldChange = −3.51, FDR-adjusted *p* < 0.001).

A longitudinal differential abundance analysis within the intervention group did not identify any taxa showing significant changes between baseline and 3 months, baseline and 6 months, or 3 and 6 months after FDR correction.

### 2.5. Exploratory Microbiota Analyses According to Supplementation Status

Because use of the nutritional supplement was optional within the intervention group and was therefore not randomized, supplementation-status comparisons were considered secondary, hypothesis-generating analyses and should not be interpreted as treatment effects. The average relative abundance of the eight most abundant bacterial taxa by supplementation status across follow-up is shown in Supplementary Figure S3.

**Figure 4:**
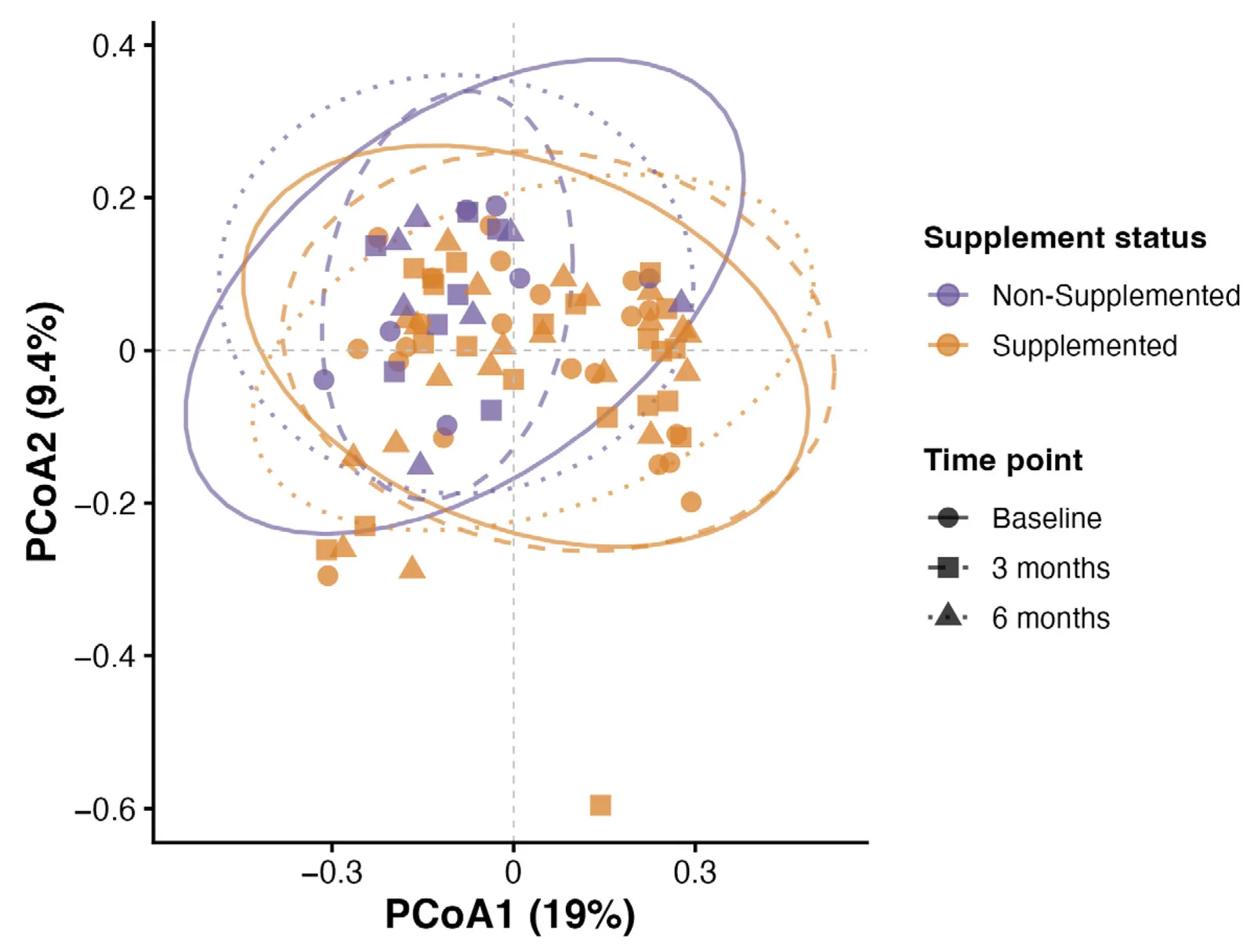
Bray–Curtis beta diversity according to supplementation status. Principal coordinates analysis (PCoA) based on Bray–Curtis dissimilarity showing gut microbial community composition in supplemented and non-supplemented participants within the intervention group across follow-up. Points represent individual samples, with colors indicating supplementation status and shapes indicating time points; 95% confidence ellipses are shown for each supplementation status–time combination. PERMANOVA with permutations restricted by participant showed no significant supplementation status × time interaction (*R*^2^ = 0.009, *F* = 0.368, *p* = 0.548). Multivariate dispersion did not differ significantly among supplementation status–time combinations (PERMDISP, *F* = 2.165, *p* = 0.067).

Principal coordinates analysis (PCoA) based on Bray–Curtis dissimilarity showed substantial overlap between supplemented and non-supplemented participants across follow-up. PERMANOVA with permutations restricted by participant did not detect a significant supplementation status × time effect on microbial community composition (*R*^2^ = 0.009, *F* = 0.368, *p* = 0.548). Multivariate dispersion did not differ significantly among supplementation status–time combinations (PERMDISP, *F* = 2.165, *p* = 0.067) (Figure 4).

**Figure 5:**
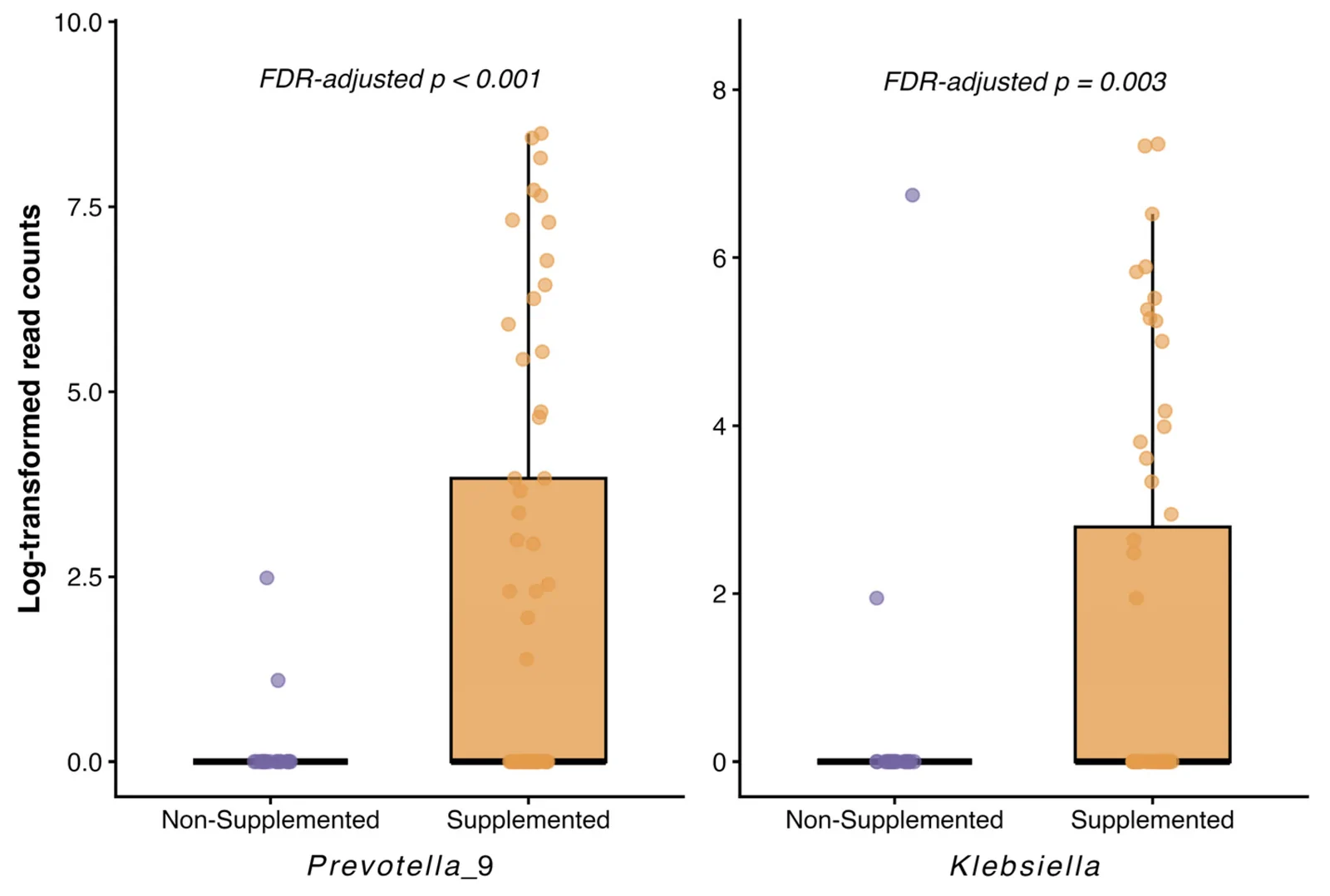
Overall differentially abundant bacterial taxa according to supplementation status across follow-up. Boxplots show the distribution of log-transformed read counts for bacterial taxa identified as significantly different between supplemented and non-supplemented participants in the overall exploratory DESeq2 analysis adjusted for sampling time. Boxes represent the interquartile range, horizontal lines indicate the median, whiskers extend to 1.5 times the interquartile range, and points represent individual samples. *p*-values were adjusted using the Benjamini–Hochberg FDR procedure, and FDR-adjusted *p*-values are displayed within each panel. These supplementation-status comparisons were exploratory and should not be interpreted as randomized treatment effects.

Exploratory supplementation analyses identified higher abundances of *Prevotella_9* (shrunken log2FoldChange = 3.626, FDR-adjusted *p* < 0.001) and *Klebsiella* (shrunken log2FoldChange = 0.932, FDR-adjusted *p* = 0.003) among supplemented participants in the overall DESeq2 analysis across follow-up (Figure 5; Supplementary Table S3A). However, time-stratified analyses showed that the differences in *Prevotella_9* and *Klebsiella* identified in the overall analysis were already evident at baseline, with higher abundances of *Prevotella_9* (shrunken log2FoldChange = 1.795, FDR-adjusted *p* < 0.001) and *Klebsiella* (shrunken log2FoldChange = 1.445, FDR-adjusted *p* < 0.001) in supplemented participants. Both taxa were absent from all seven non-supplemented participants at baseline, indicating a highly sparse and imbalanced baseline distribution (Supplementary Table S3B). No taxa differed significantly by supplementation status at 3 or 6 months after FDR correction.

In an exploratory taxonomic analysis, the genus *Bifidobacterium*, to which the heat-inactivated BPL1 strain belongs, did not differ significantly by supplementation status (FDR-adjusted *p* = 0.740). No significant between-group differences were observed at baseline, 3 months, or 6 months after FDR correction. Although mean abundance tended to be higher in supplemented participants at some time points, substantial interindividual variability was observed, and median values did not show a consistent pattern of enrichment across follow-up (Supplementary Figure S4). Given the non-viable nature of the administered BPL1 preparation, this analysis was exploratory and was not intended as a positive control or as an assessment of strain engraftment.

At the species level, *Bifidobacterium bifidum* showed higher abundance among supplemented participants in the overall exploratory analysis (shrunken log2FoldChange = 1.707, FDR-adjusted *p* < 0.001). No other species-level assignments remained significant after multiple-testing correction. Time-stratified comparisons of *Bifidobacterium bifidum* did not remain significant after FDR correction at baseline, 3 months, or 6 months. Notably, *Bifidobacterium animalis*, the species to which the administered BPL1 strain belongs, did not differ by supplementation status (FDR-adjusted *p* = 1.000) (Supplementary Table S2).

### 2.6. Exploratory Microbiota Analyses According to Supplement Adherence

Among supplemented participants, adherence-related comparisons were considered secondary, hypothesis-generating analyses. Because adherence was not randomized and may reflect participant characteristics or behaviors associated with supplement consumption, these findings should not be interpreted as causal effects of greater supplement exposure. PCoA based on Bray–Curtis dissimilarity showed substantial overlap between the low- and high-adherence groups across follow-up. PERMANOVA with permutations restricted by participant did not detect a significant adherence group × time effect on microbial community composition (*R*^2^ = 0.012, *F* = 0.377, *p* = 0.537). Multivariate dispersion did not differ significantly among adherence group–time combinations (PERMDISP, *p* = 0.792) (Supplementary Figure S5).

**Figure 6:**
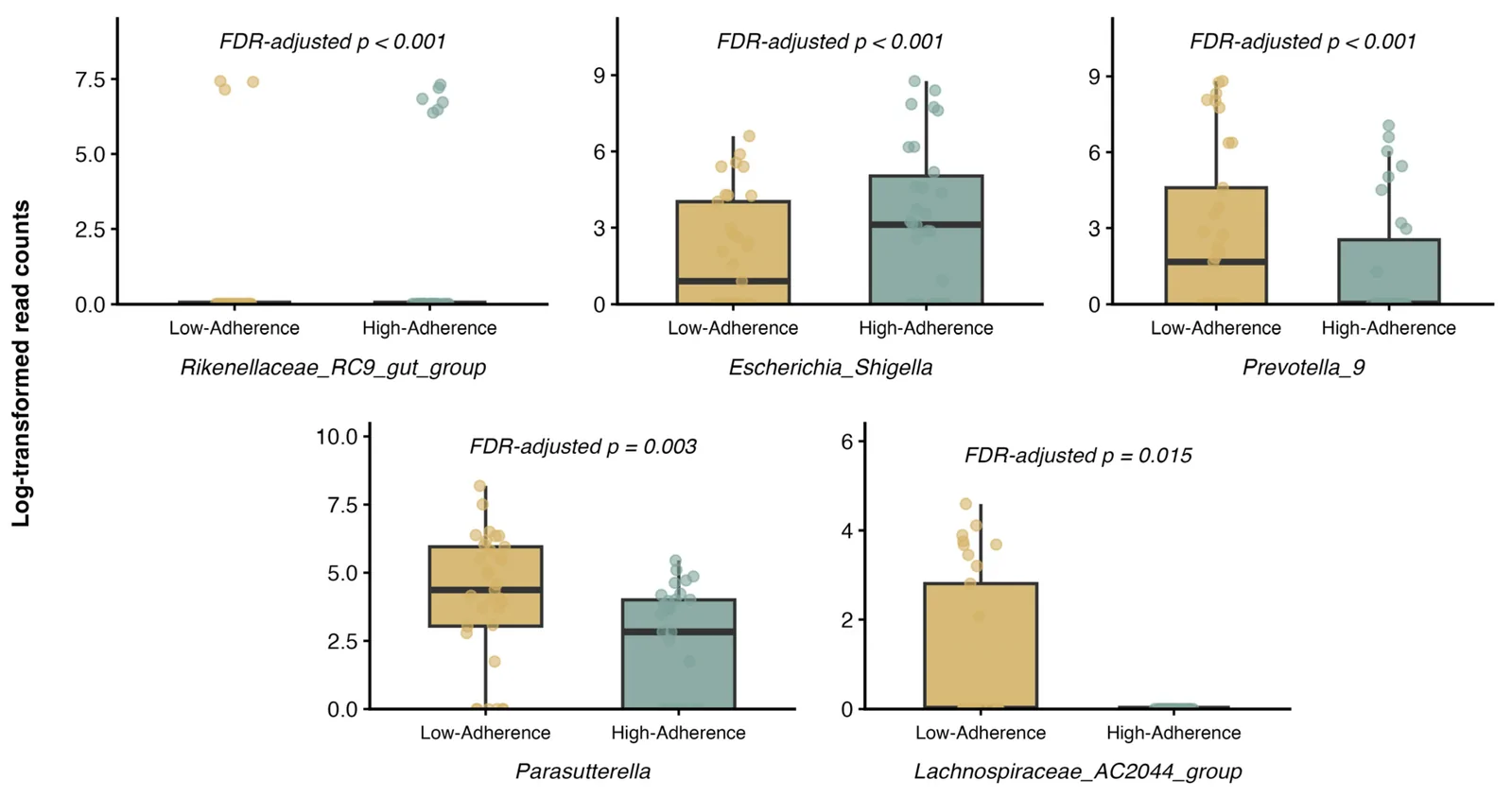
Overall differentially abundant bacterial taxa according to supplement adherence across follow-up. Boxplots show the distribution of log-transformed read counts for bacterial taxa identified as significantly different between high- and low-adherence participants in the overall exploratory DESeq2 analysis adjusted for sampling time. Boxes represent the interquartile range, horizontal lines indicate the median, whiskers extend to 1.5 times the interquartile range, and points represent individual samples. *p*-values were adjusted using the Benjamini–Hochberg false discovery rate (FDR) procedure, and FDR-adjusted *p*-values are displayed within each panel. These adherence-related comparisons were exploratory and should not be interpreted as causal effects of greater supplement exposure.

In the overall exploratory DESeq2 analysis across follow-up, adjusted for sampling time, five bacterial taxa differed significantly by adherence group after FDR correction. *Rikenellaceae_RC9_gut_group* (shrunken log2FoldChange = 0.149, FDR-adjusted *p* < 0.001) and *Escherichia-Shigella* (shrunken log2FoldChange = 2.521, FDR-adjusted *p* < 0.001) showed higher abundance in the high-adherence group, whereas *Prevotella_9* (shrunken log2FoldChange = −2.080, FDR-adjusted *p* < 0.001), *Parasutterella* (shrunken log2FoldChange = −2.572, FDR-adjusted *p* = 0.003), and *Lachnospiraceae_AC2044_group* (shrunken log2FoldChange = −3.235, FDR-adjusted *p* = 0.015) showed higher abundance in the low-adherence group (Figure 6; Supplementary Table S3A). Prevalence and zero-count frequencies for these taxa are reported in Supplementary Table S3B to facilitate interpretation of findings involving sparse distributions.

At 6 months, *Clostridium_sensu_stricto_1* was also more abundant in the high-adherence group (shrunken log2FoldChange = 0.445, FDR-adjusted *p* = 0.012), whereas no differentially abundant taxa were identified at baseline or 3 months after FDR correction.

Sensitivity analyses: Mixed-effects models accounting for repeated measurements were additionally applied to the exploratory supplementation, species-level *Bifidobacterium*, and adherence analyses. Although the direction of the observed associations was generally preserved where applicable, none of these associations remained significant after FDR correction. These findings were therefore considered sensitivity analyses and were not interpreted as confirmatory evidence.

### 2.7. Exploratory Microbiota Analyses According to Weight Loss Response

Analyses based on ≥5% weight-loss response were considered secondary, hypothesis-generating analyses and were not interpreted as causal effects of weight loss on the gut microbiota. Beta diversity was explored by weight-loss response across the three sampling time points. PCoA based on Bray–Curtis dissimilarity showed substantial overlap between participants achieving <5% and ≥5% weight loss. PERMANOVA with permutations restricted by participant did not detect a significant weight-loss response × time effect on microbial community composition (*R*^2^ = 0.007, *F* = 0.496, *p* = 0.099). Multivariate dispersion did not differ significantly among weight-loss response–time combinations (PERMDISP, *p* = 0.491). An additional exploratory analysis evaluating overall differences by subsequent weight-loss response, adjusted for sampling time, likewise showed no significant difference in microbial community composition between participants achieving <5% and ≥5% weight loss (*R*^2^ = 0.009, *F* = 1.231, *p* = 0.929; PERMDISP, *p* = 0.791) (Supplementary Figure S6).

No bacterial taxa differed significantly by subsequent weight-loss response in the overall exploratory DESeq2 analysis across the three sampling time points or in time-stratified analyses at baseline, 3 months, or 6 months after FDR correction. Mixed-effects modeling accounting for repeated measurements was additionally performed as a sensitivity analysis and likewise identified no FDR-significant associations.

## 3. Discussion

To our knowledge, this is the first study to longitudinally characterize the fecal gut microbiota using 16S rRNA gene sequencing in women with overweight or obesity and BCRL undergoing a multimodal lifestyle intervention. Despite greater reductions in body weight, BMI, fat mass, and energy intake, the intervention was not associated with detectable changes in alpha diversity or overall microbial community structure. Exploratory differential abundance analyses nevertheless identified several taxon-specific signals by study group, supplementation status, and supplement adherence. No significant microbiota differences were identified by clinically relevant weight-loss response.

Obesity is closely linked to BCRL, with higher BMI consistently identified as a risk factor for its development [18]. Reciprocal interactions between adipose tissue expansion and lymphatic dysfunction have also been described [5,6]. As previously reported in the parent trial, the multimodal intervention resulted in greater reductions in body weight, BMI, and fat mass, while women who achieved ≥5% weight loss showed greater reductions in affected limb volume at 6 months than those who achieved <5% weight loss [17]. Previous randomized dietary interventions in women with established BCRL have shown inconsistent effects on lymphedema volume: one trial reported a significant reduction in affected arm volume following weight reduction [19], whereas a subsequent trial found no significant between-group difference in lymphedema volume despite reductions in body weight [20]. In the larger WISER Survivor trial, substantial weight loss did not translate into significant improvements in BCRL outcomes [21]. Similarly, meta-analytic evidence has shown that weight-loss interventions reduced both affected and unaffected arm volumes without significantly reducing the interlimb volume difference [22]. Consistent with these findings, the greater reductions in body weight, BMI, and fat mass observed in the present study were not accompanied by significant between-group differences in lymphedema-related outcomes.

Although the groups were broadly comparable in anthropometric measures at baseline, dyslipidemia was more prevalent in the control group. This imbalance warrants consideration because gut microbiota composition has been associated with circulating lipid profiles in human population-based studies, particularly triglyceride and HDL levels and specific lipoprotein subfractions [23,24]. Moreover, the relationship between the gut microbiota and lipid metabolism is biologically plausible through several microbial pathways and metabolites, including short-chain fatty acids and secondary bile acids [25]. Therefore, the higher prevalence of dyslipidemia in the control group represents a potential source of residual confounding when interpreting taxon-specific between-group differences. Although baseline total cholesterol and triglyceride concentrations did not differ significantly between groups, these measurements may not fully capture the broader metabolic phenotype associated with dyslipidemia. Nevertheless, in a *post hoc* sensitivity analysis, the four taxa identified in the overall time-adjusted intervention-versus-control analysis remained FDR-significant and retained a consistent direction of association after additional adjustment for baseline dyslipidemia, suggesting that these exploratory associations were not solely explained by this imbalance. However, given the small number of participants with dyslipidemia (*n* = 8) and the marked between-group imbalance, residual confounding cannot be excluded.

Evidence directly linking the gut microbiota to BCRL remains scarce. Previous randomized studies in women with BCRL have evaluated synbiotic supplementation combined with calorie restriction, reporting changes in inflammatory and oxidative stress markers, quality of life, and edema-related outcomes [26,27,28]. However, these studies did not characterize gut microbial composition, and the reported improvements in quality of life and edema-related outcomes appeared to be largely attributable to calorie restriction rather than synbiotic supplementation [26]. Thus, a microbiota-mediated mechanism remains untested in BCRL. Evidence from lipedema, a distinct adipose tissue disorder that may involve secondary lymphatic alterations, is also limited [29]. A recent pilot study using 16S rRNA gene sequencing in women with lipedema found no significant differences in alpha or beta diversity compared with healthy controls, but identified differences in specific bacterial taxa [11]. Although lipedema and BCRL are distinct conditions, this pattern of taxon-specific differences without major changes in overall diversity parallels the findings of the present study.

Consistent with this emerging evidence, the present intervention did not produce detectable changes in overall gut microbial diversity or community structure despite significant reductions in body weight, BMI, and fat mass. This finding is consistent with previous intervention studies and systematic reviews showing that successful weight loss may occur with minimal or inconsistent changes in alpha and beta diversity [30,31]. Meta-analytic evidence suggests that weight loss may be associated with modest increases in alpha diversity, although with substantial between-study heterogeneity [32]. However, microbiota responses to lifestyle interventions are not uniform. In the PREDIMED-Plus trial, a 1-year intervention combining an energy-reduced Mediterranean diet with physical activity promotion was associated with increased alpha diversity and changes in specific bacterial genera [13]. Importantly, although women achieving ≥5% weight loss in the parent trial showed greater reductions in affected limb volume at 6 months than those achieving <5% weight loss [17], this clinical response was not accompanied by detectable longitudinal differences in overall microbial community composition or FDR-significant differential taxonomic abundance by weight-loss response in the present microbiota substudy. This absence of a detectable microbiota signal limits the support provided by the present data for the specific hypothesis that the greater reduction in affected limb volume associated with clinically relevant weight loss is accompanied by measurable restructuring of the fecal bacterial community. However, it does not exclude a potential role of the gut microbiota within the broader biological interplay linking obesity, inflammation, and lymphatic dysfunction. Gut microbiota alterations have been linked to obesity-associated metabolic and inflammatory disturbances [7,9,10], whereas adipose tissue expansion and obesity-related inflammation can adversely affect lymphatic function [5,6]. Weight loss may influence lymphedema through several mechanisms related to reduced adipose tissue burden, including decreased mechanical stress on the lymphatic vasculature and attenuation of obesity-associated inflammatory processes that can impair lymphatic function [5,6], without necessarily producing detectable changes in fecal bacterial taxonomic composition. Moreover, 16S rRNA gene sequencing provides limited strain-level resolution and does not directly characterize microbial functional or metabolic profiles; therefore, functionally relevant microbiome changes could occur in the absence of detectable taxonomic restructuring [33]. These findings should also be interpreted in light of the exploratory nature and limited sample size of the weight-loss-response analysis (≥5% weight loss, *n* = 16; <5%, *n* = 32). The microbiota substudy was not designed or powered specifically to detect differences between these secondary subgroups. The small and unbalanced group sizes, substantial interindividual microbiota variability, and multiple-testing correction in taxon-level analyses therefore limited sensitivity to modest associations. Accordingly, the absence of statistically significant microbiota differences should be interpreted as a lack of detectable taxonomic differences under the present design rather than evidence that microbiota-related mechanisms are absent. Differences in intervention duration, the magnitude of dietary change and weight loss, participant characteristics, sample size, and microbiota methodology may partly explain these divergent findings.

Exploratory differential abundance analyses revealed several taxon-specific patterns despite the absence of broad community-level restructuring. In the overall analysis across follow-up, Barnesiellaceae and Desulfovibrionaceae showed higher abundance in the intervention group, whereas *Prevotella_7* and *Lachnospiraceae_UCG_010* were more abundant in controls. The higher abundance of *Prevotella_7* in controls was already evident at baseline and was again detected at 6 months, suggesting that this difference may partly reflect a pre-existing microbial configuration rather than an intervention-induced effect. Pretreatment *Prevotella*-to-*Bacteroides* profiles have previously been associated with subsequent weight-loss maintenance [34], underscoring the importance of baseline microbiota profiles when interpreting intervention studies. Barnesiellaceae has previously been identified among microbial markers of normal-weight adolescents [35], although direct extrapolation of this age-specific, cross-sectional finding to the present cohort is limited. Desulfovibrionaceae also illustrates the context-dependent nature of taxonomic associations: although *Desulfovibrio* species have been linked to several inflammatory and metabolic conditions [36], a 6-month lifestyle-induced weight-loss intervention reported increased Desulfovibrionaceae alongside improvements in BMI, insulin sensitivity, and inflammatory markers [37]. Associations involving Lachnospiraceae are likewise heterogeneous across obesity and metabolic disorders [38]. At 6 months, *Escherichia-Shigella* was more abundant in controls, a genus group previously reported at higher abundance in adults with obesity [39]; however, its absence from the weight-loss-response analysis argues against a simple relationship with the greater weight reduction observed in the intervention group. In addition, the corresponding effect-size estimate was markedly attenuated after shrinkage, limiting interpretation of the magnitude of this difference. Finally, the participant-blocked longitudinal analysis yielded FDR-significant group × time tests for Izemoplasmatales and *Frisingicoccus* for changes from baseline to 3 months. However, the corresponding effect-size estimates were markedly attenuated after shrinkage, limiting interpretation of the magnitude and direction of these associations. No FDR-significant group × time differences were detected from baseline to 6 months. Evidence regarding the metabolic relevance of these taxa remains limited. Izemoplasmatales was more abundant in individuals characterized by a muscle insulin-resistance phenotype than in those with liver insulin resistance [40]. *Frisingicoccus* has been reported to be enriched in women with breast cancer and obesity compared with those without obesity [41]. These observations are of potential interest in the context of the divergent adiposity trajectories between groups, but they do not establish a relationship between either taxon and changes in adiposity. Overall, the findings identify several taxon-specific and temporal patterns, but their biological relevance and relationship to the intervention remain uncertain.

Exploratory analyses by supplementation status identified additional taxon-specific differences. In the overall analysis, *Prevotella_9* and *Klebsiella* were more abundant among supplemented participants. Importantly, the differences in *Prevotella_9* and *Klebsiella* were already evident at baseline, whereas no significant differences were detected at 3 or 6 months, suggesting that pre-existing microbial differences may have contributed to the overall associations. Previous evidence from the PREDIMED-Plus cohort has associated greater *Prevotella_9* abundance with greater weight loss following a 12-month lifestyle intervention [42], although this observation does not establish a similar role in the present cohort. In a randomized trial of a multicomponent product containing heat-inactivated BPL1, inulin, and omega-3 fatty acids, the intervention reduced pulse pressure in women, and *Prevotella 9* ASV0283 abundance was negatively correlated with pulse pressure [43], suggesting that *Prevotella_9*-related taxa may be relevant in specific metabolic contexts. The contrasting patterns observed for *Prevotella_9* and *Prevotella_7* should not be considered contradictory, as they represent distinct taxonomic assignments with potentially different ecological associations. Importantly, *Klebsiella* was already more abundant among supplemented participants at baseline, indicating a pre-existing taxonomic difference rather than an effect of supplementation. Its overall effect-size estimate was also substantially attenuated after shrinkage, consistent with its sparse distribution, particularly among non-supplemented participants. Although some *Klebsiella* taxa have recognized pathogenic potential [44], no corresponding adverse clinical signal was apparent during follow-up.

At the genus level, *Bifidobacterium* did not differ by supplementation status. At the species level, *Bifidobacterium animalis,* the species to which the administered heat-inactivated BPL1 strain belongs, also showed no significant difference, whereas *Bifidobacterium bifidum* was more abundant among supplemented participants. Importantly, the absence of an increase in *Bifidobacterium* should not be interpreted as evidence of a lack of biological effect of supplementation. Because BPL1 was administered in a heat-inactivated form, an increase in the relative abundance of the administered organism or its engraftment was not expected, and these analyses were exploratory rather than intended as a positive control. Moreover, species-level inference from 16S rRNA gene sequencing is limited, particularly for closely related *Bifidobacterium* species. Previous randomized evidence has demonstrated metabolic effects of heat-inactivated BPL1 [16]. The multicomponent trial discussed above also reported changes in gut microbiota composition alongside reductions in insulin, HOMA-IR, and, in women, pulse pressure [43]. Cross-feeding interactions among bifidobacteria are well described, whereby nutrient availability and microbial metabolites can influence the trophic niches of resident bifidobacterial populations [45]. Although the administered BPL1 was heat-inactivated and therefore would not be expected to participate directly in metabolic cross-feeding, preclinical evidence indicates that BPL1-derived lipoteichoic acid retains biological activity [46]. Together with the other bioactive components of the supplement, these effects could potentially modify the intestinal environment and indirectly influence resident bifidobacteria; however, the present data do not establish such a mechanism. Accordingly, the *Bifidobacterium bifidum* association should be considered exploratory and requires further investigation.

Further exploratory analyses by supplement adherence identified taxon-specific differences despite the absence of detectable changes in overall community structure. In the overall analysis, *Rikenellaceae_RC9_gut_group* and *Escherichia-Shigella* were more abundant among participants with high adherence, whereas *Prevotella_9*, *Parasutterella*, and *Lachnospiraceae_AC2044_group* were more abundant among those with low adherence. Higher abundance of the Rikenellaceae family has been associated with lower visceral adiposity and a healthier metabolic profile [47], although this family-level observation cannot be directly extrapolated to *Rikenellaceae_RC9_gut_group*. Moreover, this taxon was detected in relatively few samples, and its effect-size estimate was almost abolished after shrinkage; this association should therefore be interpreted with particular caution. Conversely, *Parasutterella,* which was more abundant in the low-adherence group, has been associated with higher BMI, type 2 diabetes, and dietary carbohydrate intake and has been reported to decrease during weight loss [48]. The enrichment of *Escherichia-Shigella* among participants with high adherence should likewise be interpreted in context. Although this taxon has been reported at higher abundance in adults with obesity [39], greater baseline *Escherichia-Shigella* abundance was associated with greater reductions in weight and BMI following a fiber-rich dietary intervention [49], highlighting its context-dependent association with metabolic outcomes. Similarly, the higher abundance of *Prevotella_9* in the low-adherence group contrasts with its enrichment among supplemented participants, indicating that supplementation status did not translate into a simple adherence-related gradient. *Lachnospiraceae_AC2044_group* was more abundant in the low-adherence group; increases in this taxon have previously been associated with a trend toward greater HOMA-IR improvement following dietary intervention [50], again illustrating the context-dependent nature of these associations. No significant adherence-related taxa were detected at baseline or 3 months, whereas at 6 months *Clostridium_sensu_stricto_1* was more abundant among participants with high adherence. This 6-month association was also based on a small number of positive samples and was markedly attenuated after shrinkage. A similar increase in *Clostridium_sensu_stricto_1* has been reported following probiotic supplementation in healthy older adults [51], although differences in the study populations and interventions preclude direct comparison. Overall, these findings suggest that supplement adherence may be associated with subtle and taxon-specific microbial patterns rather than broad community-level changes, and their biological relevance warrants further investigation. More broadly, the taxon-specific associations identified in the exploratory DESeq2 analyses were not confirmed after FDR correction in mixed-effects sensitivity models accounting for repeated measurements. This lack of confirmation limits the robustness of these findings and supports their interpretation as hypothesis-generating rather than confirmatory evidence. Several taxon-level associations involved sparse count distributions and large unshrunken effect-size estimates. Accordingly, log2 fold-change shrinkage was applied to FDR-significant findings, resulting in substantial attenuation of some estimates. Together with the observed prevalence and zero-count distributions, these findings indicate that effect-size magnitudes should be interpreted cautiously, particularly for low-prevalence taxa, even when the corresponding differential-abundance tests remained significant after FDR correction.

The present study has several limitations that should be considered when interpreting these findings. First, this exploratory microbiota substudy had a modest sample size, with smaller subgroups for supplementation status, supplement adherence, and clinically relevant weight loss, limiting statistical power, particularly for secondary subgroup comparisons and the detection of modest microbiota associations, and increasing sensitivity to interindividual variability. The requirement for complete, quality-controlled fecal samples at all three time points reduced the analytical microbiota cohort relative to the parent randomized trial, which may limit the generalizability of the findings to the full trial population. Supplement use was optional within the intervention group, and adherence varied among participants; therefore, supplementation- and adherence-based comparisons were not randomized and may be subject to self-selection, baseline imbalance, and residual behavioral or dietary confounding. Indeed, the baseline differences observed for *Prevotella_9* and *Klebsiella* between supplemented and non-supplemented participants further limit attribution of subsequent between-group differences to supplementation. Accordingly, these analyses should be considered hypothesis-generating rather than evidence of supplementation or adherence effects. Because the nutritional supplement contained multiple bioactive components, the observed microbial patterns cannot be attributed specifically to heat-inactivated BPL1. Weight-loss-response analyses were also exploratory and do not support causal inference. The microbiota substudy was exploratory and did not include a single preregistered confirmatory microbiota endpoint. Given the number of complementary, secondary, and subgroup analyses performed, chance findings despite within-analysis FDR correction cannot be excluded. Across the genus/nearest-resolved-level differential-abundance analyses, 110–130 taxa were tested per analysis set, of which 0–5 were FDR-significant, yielding 19 FDR-significant findings in total across all analysis sets. One additional FDR-significant species-level finding was identified in the exploratory *Bifidobacterium* analysis. As a descriptive approximation, applying the 5% FDR threshold to the number of significant findings would correspond to ≤0.25 potential false discoveries within an individual analysis set and approximately one across the 20 reported significant findings. However, this should not be interpreted as a formal estimate of the number of false discoveries, because Benjamini–Hochberg controls the expected false discovery proportion within each analysis set. FDR control was applied separately within each analysis set and does not account for multiplicity across the multiple complementary, secondary, and subgroup analyses performed. To provide a clearer analytical hierarchy, inferential priority was assigned to the participant-blocked longitudinal differential-abundance models for the main randomized intervention-versus-control comparison, whereas the overall time-adjusted and time-stratified analyses were considered complementary and the supplementation-, adherence-, and weight-loss-related analyses were considered secondary and hypothesis-generating. Baseline clinical differences, particularly the higher prevalence of dyslipidemia in the control group, as well as pre-existing taxonomic differences, should also be considered when interpreting between-group microbial patterns, as they may contribute to residual confounding and complicate attribution of subsequent differences to the intervention. Finally, species-level findings derived from multi-region 16S rRNA gene sequencing should be considered exploratory, particularly for closely related *Bifidobacterium* taxa, and strain-level resolution of BPL1 was not possible.

Nevertheless, the study has important strengths, including longitudinal gut microbiota characterization at three time points within a randomized lifestyle intervention in a population for which direct microbiome evidence remains scarce. Detailed clinical, anthropometric, dietary, and lymphedema-related phenotyping provided a well-characterized clinical context for interpreting the microbial findings. Analytical approaches accounted for repeated measurements and multivariate dispersion and incorporated multiple-testing correction and complementary mixed-effects sensitivity analyses. Exploratory analyses of supplementation, adherence, and clinically relevant weight loss provided additional perspectives on taxon-specific microbiota patterns.

## 4. Materials and Methods

### 4.1. Study Design and Participants

This gut microbiota substudy was conducted within the framework of a previously published randomized pilot trial performed at the Regional Hospital of Málaga between June 2021 and June 2023 (ClinicalTrials.gov identifier: NCT04974268). The parent study used an open-label, two-arm parallel design and enrolled women aged 18–79 years with BCRL and a BMI of 25–40 kg/m^2^. Participants were randomized in a 1:1 ratio to a structured lifestyle intervention or a standard-care control group. Detailed eligibility criteria and randomization procedures have been reported previously [17].

**Figure 7:**
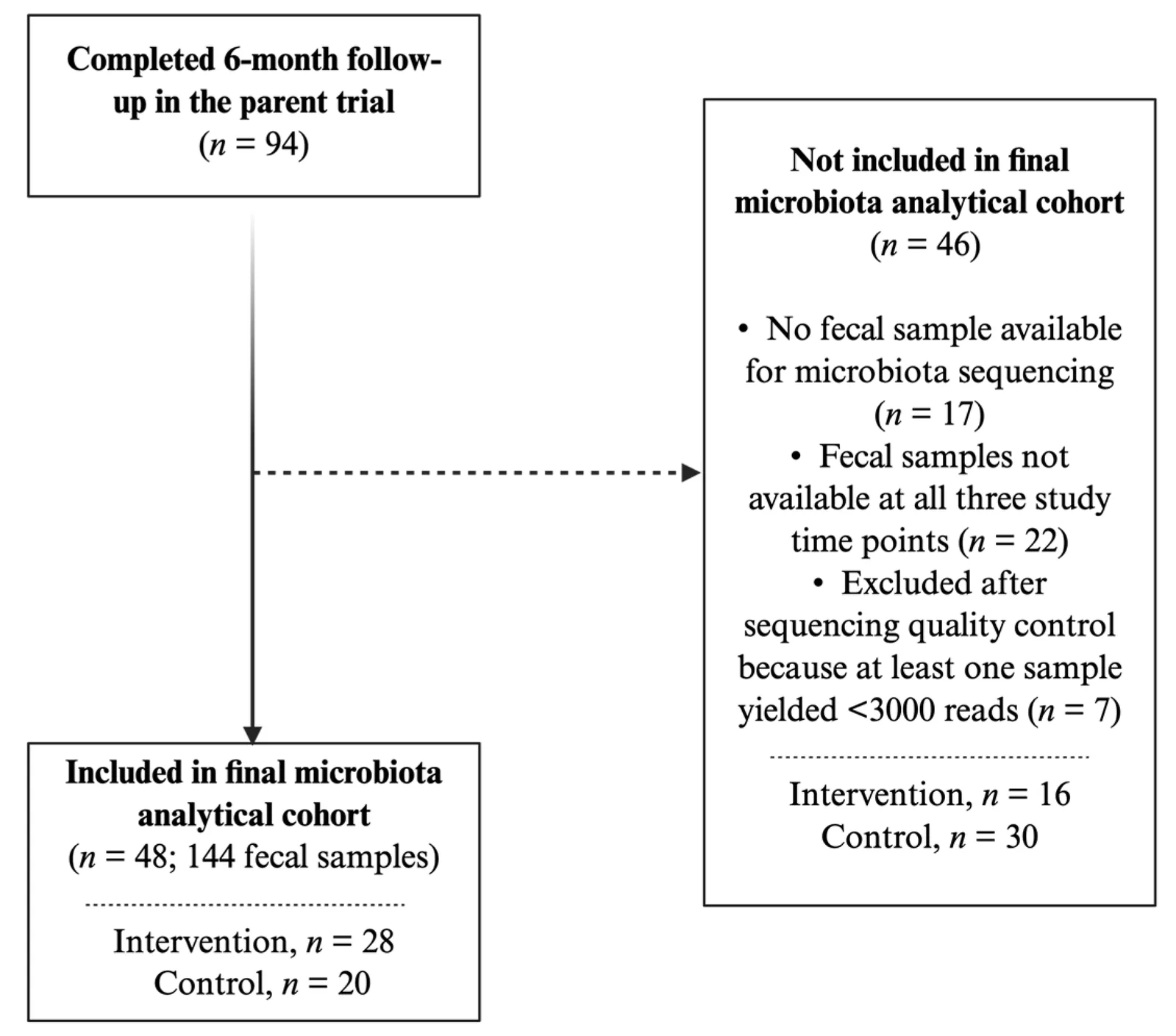
Flowchart of participant selection for the gut microbiota substudy. The flowchart summarizes the selection of the final microbiota analytical cohort from participants who completed the 6-month follow-up of the parent trial. Participants included in the final analytical cohort had fecal samples available at baseline, 3 months, and 6 months, with all samples yielding ≥3000 reads after quality filtering. Reasons for non-inclusion in the final microbiota cohort are shown. Created with BioRender.com.

For inclusion in the present microbiota analysis, participants were required to provide fecal samples at baseline, 3 months, and 6 months, with all three samples yielding at least 3000 reads after quality filtering. The final analytical sample comprised 48 participants, including 28 women from the intervention group (60.3 ± 12.0 years) and 20 from the control group (59.1 ± 11.6 years). No significant baseline differences were observed between participants included in the final microbiota cohort (*n* = 48) and trial completers not included in the microbiota analysis (*n* = 46) with respect to age, anthropometric and body-composition measures, lymphedema-related variables, dietary characteristics, and dyslipidemia (all *p* ≥ 0.14). Participant selection for the microbiota substudy is summarized in Figure 7. All participants provided written informed consent to participate in the study. Ethical approval was granted by the Provincial Research Ethics Committee of Málaga (EJERDIETLINF; 26 March 2020), and the study was conducted in accordance with the Declaration of Helsinki.

### 4.2. Lifestyle Intervention and Exploratory Participant Classifications

The intervention group participated in a structured lifestyle program combining supervised resistance and aerobic exercise with an energy-restricted Mediterranean-style diet aimed at promoting clinically relevant weight loss. Participants allocated to the control group received standard dietary and physical activity recommendations without supervised exercise. Lymphedema management, including manual lymphatic drainage and compression garments when clinically indicated, was provided according to usual care in both groups. Full details of the intervention protocol have been reported previously [17].

As part of the nutritional intervention, participants in the intervention group could voluntarily consume one daily serving of Bi1 Bificare^®^ (Adventia Pharma S.L., Las Palmas de Gran Canaria, Spain), a high-protein oral nutritional supplement containing extra virgin olive oil, leucine, omega-3 fatty acids (eicosapentaenoic acid and docosahexaenoic acid), and *Bifidobacterium animalis* subsp. *lactis* CECT 8145 (BPL1; 3.3 × 10^9^ colony-forming units [CFU] per serving), provided in a heat-inactivated form.

The main microbiota comparison evaluated longitudinal differences between the randomized intervention and control groups across baseline, 3 months, and 6 months. Because the microbiota substudy was exploratory, no single microbiota outcome was designated as a preregistered confirmatory primary endpoint. Community-level diversity and taxon-level differential abundance analyses were therefore considered complementary components of an exploratory characterization. Analyses by optional supplementation status, supplement adherence, and ≥5% weight-loss response were considered secondary, hypothesis-generating analyses and were not interpreted as randomized treatment comparisons.

Because supplement use was optional within the intervention group, secondary exploratory analyses were conducted by supplementation status. Of the 28 participants in the intervention group, 21 consumed the supplement during follow-up, whereas 7 did not. Among participants who consumed the optional supplement, adherence was categorized based on the observed median number of servings consumed during the 6-month intervention. Participants consuming >92 servings were classified as having high adherence (*n* = 10), whereas those consuming ≤92 servings were classified as having low adherence (*n* = 11). The cutoff was used for exploratory stratification by supplement adherence.

Finally, exploratory analyses were performed by weight-loss response. Consistent with the parent trial, clinically relevant weight loss was defined as a reduction of ≥5% from baseline body weight achieved during the study [17]. Participants were therefore classified as achieving clinically relevant weight loss (≥5% weight loss, *n* = 16) or not achieving clinically relevant weight loss (<5% weight loss, *n* = 32). Complementary exploratory comparisons were also performed at individual sampling time points (baseline, 3 months, and 6 months) to characterize time-point-specific differences in gut microbiota composition.

### 4.3. Clinical and Anthropometric Assessments

Clinical and demographic information was collected at baseline, including age, smoking status, affected limb, cancer stage, previous breast cancer treatments (mastectomy, axillary lymph node dissection, sentinel lymph node biopsy, chemotherapy, and radiotherapy), and relevant comorbidities, including hypertension, diabetes mellitus, and dyslipidemia. Hypertension was defined as systolic blood pressure ≥140 mmHg and/or diastolic blood pressure ≥90 mmHg, or a documented diagnosis requiring antihypertensive treatment [52]. Dyslipidemia was recorded based on a documented clinical diagnosis and/or current lipid-lowering treatment [53]. Physical activity was assessed using the short version of the International Physical Activity Questionnaire (IPAQ), with results expressed as metabolic equivalent of task minutes per week (MET-min/week).

Anthropometric and body composition assessments were performed at baseline, 3 months, and 6 months. BMI was calculated using the Quetelet formula: weight (kg)/height (m^2^) [54]. Waist circumference was measured according to standardized anthropometric procedures. Body composition was assessed by bioelectrical impedance analysis (BIA) using a TANITA MC980MA analyzer (Tanita Corporation, Tokyo, Japan), with participants assessed in a standing position according to standardized procedures. Body weight, fat mass, fat-free mass, and phase angle were obtained from the analyzer, and measurement reports were generated using Dietowin software (version 8.0; Dietowin S.L., La Garriga, Barcelona, Spain).

Lymphedema-related measurements were obtained at baseline, 3 months, and 6 months. Limb circumference and volume were assessed using the SERMEF calculator (Spanish Society of Rehabilitation and Physical Medicine [SERMEF], Madrid, Spain), which estimates limb volume from circumferential measurements using the truncated-cone method [55]. Absolute and relative interlimb volume differences were derived from the estimated volumes of the affected and unaffected limbs.

Blood samples were collected after an overnight fast at baseline, 3 months, and 6 months. C-reactive protein, fasting glucose, total cholesterol, and triglycerides were measured using standardized automated laboratory methods.

### 4.4. Dietary Intake and Mediterranean Diet Adherence

Dietary intake was assessed at baseline, 3 months, and 6 months using a 4-day prospective dietary record comprising three weekdays and one weekend day. Participants recorded all foods and beverages consumed, with portion sizes estimated using household measures. Dietary records were analyzed using Dietstat^®^ (FIMABIS, Málaga, Spain; institutional version 2016) to estimate daily energy and nutrient intake [17]. Adherence to the Mediterranean diet was evaluated using the 17-item PREDIMED-Plus questionnaire, which provides a score ranging from 0 to 17, with higher scores indicating greater adherence to the Mediterranean dietary pattern [17].

### 4.5. Fecal Sample Collection and 16S rRNA Gene Sequencing

Fecal samples were collected during the scheduled study assessments, yielding 144 samples for microbiota analysis. Participants were provided with sterile, hermetically sealed containers and instructed to collect the fecal sample either the day before or on the day of the study visit. Following collection, samples were stored in household freezers until delivery and transported on ice to minimize temperature fluctuations. Upon receipt, fecal samples were transferred to the Biobank of the Public Health System of Andalusia (SSPA) and stored at −80 °C until DNA extraction and subsequent microbiota analysis.

Genomic DNA was extracted from 200 mg of fecal material using the QIAamp DNA Stool Mini Kit (Qiagen, Hilden, Germany) according to the manufacturer’s instructions. DNA concentration was determined by absorbance at 260 nm, and purity was assessed using the A260/A280 ratio with a NanoDrop spectrophotometer (NanoDrop Technologies, Wilmington, DE, USA). The bacterial 16S rRNA gene was amplified using the Ion 16S Metagenomics Kit (Thermo Fisher Scientific, Waltham, MA, USA), using two separate primer pools targeting the V2, V4, and V8 regions and the V3, V6–V7, and V9 regions, respectively. Amplicons generated from both reactions were subsequently combined for library preparation. Barcoded libraries were prepared using the Ion Plus Fragment Library Kit (Thermo Fisher Scientific) and pooled for automated template preparation using the Ion Chef System (Thermo Fisher Scientific). Sequencing was subsequently performed on the Ion S5 platform (Thermo Fisher Scientific). Base calling and run demultiplexing were performed using Torrent Suite software (version 5.4.0; Thermo Fisher Scientific). Synthetic reference microbial communities and negative controls were included during PCR amplification and library preparation. Samples were sequenced across two Ion S5 runs, with all available longitudinal samples from the same participant sequenced within the same run.

### 4.6. Bioinformatic Processing and Microbiota Analysis

Sequence reads were processed using QIIME 2 (version 2023.7) [56]. Single-end reads were subjected to quality filtering, denoising, and chimera removal using the DADA2 algorithm implemented in the q2-dada2 plugin [57], generating amplicon sequence variants (ASVs). Reads were truncated at 150 bp during denoising. Taxonomic assignment was performed using the Greengenes2 reference database [58], and non-bacterial, mitochondrial, and chloroplast sequences were excluded during bioinformatic processing. The resulting feature-abundance and taxonomic annotation tables were used for downstream microbiota analyses. Samples yielding <3000 reads after quality filtering were excluded. For diversity analyses, samples were rarefied to 3000 reads per sample, maximizing retention of complete longitudinal cases while maintaining adequate sequencing coverage. Taxonomic analyses were conducted at the genus level or, when genus-level assignment was not available, at the nearest resolved taxonomic level.

Sequencing coverage was assessed using Good’s coverage index. Alpha diversity was characterized using the Chao1, Shannon, Simpson, and inverse Simpson indices and analyzed longitudinally using linear mixed-effects models including the grouping variable, time, and their interaction as fixed effects and participant as a random intercept. Interaction effects were assessed by likelihood-ratio tests, with Holm correction across the four indices. Beta diversity was assessed using Bray–Curtis dissimilarity from rarefied abundances and visualized by PCoA. Longitudinal community-level differences were evaluated by PERMANOVA with 9999 permutations restricted by participant, with PERMDISP used to assess homogeneity of multivariate dispersion. The same diversity framework was applied to comparisons by study group and the exploratory classifications defined above. Microbiota visualizations were generated in R using vegan (version 2.7-5) and ggplot2 (version 4.0.3).

Differential abundance analyses were performed using DESeq2 (version 1.50.2) [59] on non-rarefied counts. Taxa present in at least 10% of samples in the corresponding analysis set were retained, and size factors were estimated using the *poscounts* method. Of the 255 taxa identified in the dataset, 119 were retained in the overall analysis; 115, 123, and 130 in the baseline, 3-month, and 6-month analysis sets, respectively. In the supplementation analyses, 120 taxa were retained overall and 124, 121, and 130 at baseline, 3 months, and 6 months, respectively; the corresponding numbers for the adherence analyses were 119, 110, 116, and 119. The weight-loss-response analyses retained 119 taxa overall and 115, 123, and 130 at the three respective time points. *p*-values were adjusted using the Benjamini–Hochberg FDR, with FDR-adjusted *p* < 0.05 considered statistically significant. For FDR-significant taxa, log2 fold-change estimates were additionally stabilized using the DESeq2 *lfcShrink* function with the normal prior; reported effect sizes for these taxa are shrunken log2 fold changes unless otherwise stated, whereas *p*-values and FDR-adjusted *p*-values correspond to the original DESeq2 hypothesis tests. For the participant-blocked group × time models, the longitudinal design was supplied as an explicit model matrix with betaPrior = FALSE, and shrinkage was applied to the corresponding interaction coefficient using the *coef* argument of *lfcShrink* with the normal prior. Prevalence and zero-count frequencies were also summarized for FDR-significant taxa to facilitate interpretation of findings involving sparse count distributions. The same general framework was applied to analyses by study group, supplementation status, supplement adherence, and weight-loss response. Analyses by supplementation status, supplement adherence, and weight-loss response were considered secondary, hypothesis-generating analyses, as described above. Overall exploratory models included sampling time as a covariate, with complementary analyses conducted separately at baseline, 3 months, and 6 months. For nested supplementation and adherence analyses, size factors were estimated in the corresponding parent analysis set before subsetting.

For the main randomized intervention-versus-control comparison, longitudinal differential abundance was additionally evaluated using participant-blocked DESeq2 models including participant effects nested within study group and group × time interactions. After application of the same ≥10% prevalence criterion, 119 taxa were retained in these longitudinal models. Because these models directly evaluated between-group differences in longitudinal trajectories while accounting for repeated measurements, they were given inferential priority over the complementary overall time-adjusted and time-stratified differential abundance analyses. Contrasts estimated between-group differences in changes from baseline to 3 months and from baseline to 6 months. As a sensitivity analysis, the main intervention-versus-control differential-abundance analyses were repeated using a more stringent prevalence threshold of ≥20% of samples. This threshold retained 94 of the 255 taxa in the overall analysis set and 95, 93, and 95 taxa in the baseline, 3-month, and 6-month analysis sets, respectively, and was used to assess the robustness of the main intervention-versus-control findings to the exclusion of lower-prevalence taxa. To explore potential confounding by the baseline imbalance in dyslipidemia, the overall time-adjusted intervention-versus-control DESeq2 analysis was additionally repeated as a post hoc sensitivity analysis including baseline dyslipidemia as a covariate (~Time + Dyslipidemia + Group). Because dyslipidemia status was unavailable for one participant, both the adjusted model and the corresponding unadjusted model (~Time + Group) were fitted in the same 47 participants (141 samples). Within-intervention longitudinal changes were evaluated using participant-adjusted models including participant and time. Overall differential abundance findings were further examined using MaAsLin2 (version 1.24.1), with total-sum scaled and log-transformed abundances, sampling time as a fixed effect, and participant as a random intercept. Weight-loss response was additionally evaluated by exploratory PERMANOVA adjusted for sampling time, with permutations performed at the participant level.

Because the nutritional supplement contained BPL1, an exploratory species-level *Bifidobacterium* analysis was performed by supplementation status without prevalence filtering. Species-level counts were analyzed using DESeq2 with normalization factors derived from the corresponding supplementation analysis and complemented by a mixed-effects sensitivity analysis accounting for repeated measurements. These findings were interpreted cautiously given the limited species- and strain-level resolution of 16S rRNA gene sequencing.

### 4.7. Statistical Analysis

Statistical analyses and data processing were performed using R version 4.5.1 (R Foundation for Statistical Computing, Vienna, Austria) within RStudio version 2026.07.1+147 (Posit Software, PBC, Boston, MA, USA). Normality of continuous variables was assessed using the Shapiro–Wilk test. Continuous variables are presented as mean ± standard deviation (SD) or median and interquartile range (IQR), depending on data distribution, and categorical variables as frequencies and percentages. Baseline characteristics were compared between the intervention and control groups using Welch’s *t*-test or the Mann–Whitney *U* test for continuous variables, as appropriate, and Pearson’s chi-squared (χ^2^) test or Fisher’s exact test for categorical variables, as appropriate. Percentages were calculated based on available data for each variable.

Changes from baseline to 3 and 6 months were calculated for each participant as the follow-up value minus the corresponding baseline value. Between-group differences in these changes were assessed using Welch’s *t*-test or the Mann–Whitney *U* test, according to data distribution. Longitudinal changes in clinical and nutritional outcomes were analyzed using linear mixed-effects models including group, time, and the group × time interaction as fixed effects and participant as a random intercept. Analyses were based on available data for each outcome and time point.

Between-group differences in alpha-diversity indices at baseline, 3 months, and 6 months, as well as changes from baseline to 3 months (Δ0–3) and 6 months (Δ0–6), were assessed using the Mann–Whitney *U* test. Longitudinal modeling and multiple-testing correction for microbiota outcomes are described in the preceding section. All tests were two-sided, and statistical significance was set at *p* < 0.05, except for differential abundance analyses, for which statistical significance was defined as an FDR-adjusted *p* < 0.05.

## 5. Conclusions

To our knowledge, this is the first study to longitudinally characterize the gut microbiota in women with overweight or obesity and BCRL undergoing a multimodal lifestyle intervention. Despite greater reductions in body weight, BMI, and fat mass, the intervention was not associated with detectable changes in overall microbial diversity or community structure. Nevertheless, exploratory analyses identified distinct taxon-specific and temporal patterns by study group, supplementation status, and supplement adherence, including FDR-significant group × time tests for Izemoplasmatales and *Frisingicoccus* with markedly attenuated effect-size estimates, as well as supplementation- and adherence-related differences in several bacterial taxa. No significant microbiota differences were observed by clinically relevant weight-loss response. A tentative species-level association involving *Bifidobacterium bifidum* was observed among supplemented participants, whereas *Bifidobacterium* at the genus level and *Bifidobacterium animalis* at the species level did not differ by supplementation status. Overall, these findings provide no evidence of broad community-level restructuring but identify several exploratory taxon-specific signals that require independent confirmation. Larger longitudinal studies using strain-resolved sequencing and functional approaches are needed to clarify their biological and clinical relevance in BCRL.

## Figures and Tables

**Table 1:** Baseline characteristics of participants included in the gut microbiota substudy.

Characteristic	Overall Population (*n* = 48)	Intervention Group (*n* = 28)	Control Group (*n* = 20)	*p*-Value
Demographic and lifestyle characteristics				
Age, years	59.8 ± 11.7	60.3 ± 12.0	59.1 ± 11.6	0.732
Current smoking, *n* (%)	1 (2.1%)	1 (3.6%)	0 (0.0%)	1.000
Physical activity, MET-min/week	1587.0 (866.2–3165.8)	1288.0 (634.0–3365.2)	1848.8 (1309.5–2923.5)	0.362
Cancer-related characteristics				
Cancer stage, *n* (%)				0.521
Stage I	6 (15.8%)	5 (20.0%)	1 (7.7%)	
Stage II	9 (23.7%)	5 (20.0%)	4 (30.8%)	
Stage III	20 (52.6%)	12 (48.0%)	8 (61.5%)	
Stage IV	3 (7.9%)	3 (12.0%)	0 (0.0%)	
Mastectomy, *n* (%)	30 (62.5%)	15 (53.6%)	15 (75.0%)	0.226
Axillary lymph node dissection, *n* (%)	39 (81.2%)	23 (82.1%)	16 (80.0%)	1.000
Sentinel lymph node biopsy, *n* (%)	9 (18.8%)	4 (14.3%)	5 (25.0%)	0.460
Chemotherapy, *n* (%)	40 (90.9%)	22 (88.0%)	18 (94.7%)	0.622
Radiotherapy, *n* (%)	42 (89.4%)	26 (92.9%)	16 (84.2%)	0.381
Comorbidities				
Hypertension, *n* (%)	17 (35.4%)	9 (32.1%)	8 (40.0%)	0.760
Diabetes mellitus, *n* (%)	4 (8.3%)	1 (3.6%)	3 (15.0%)	0.294
Dyslipidemia, *n* (%)	8 (17.0%)	1 (3.7%)	7 (35.0%)	**0.007**
Anthropometry and body composition				
Body weight, kg	73.1 (66.2–80.4)	73.0 (65.7–80.4)	73.1 (70.0–80.3)	0.427
BMI, kg/m^2^	29.5 (26.8–31.1)	29.7 (27.0–31.0)	29.3 (26.4–31.3)	0.638
Waist circumference, cm	90.6 ± 11.9	91.6 ± 13.1	89.2 ± 10.3	0.496
Fat mass, kg	26.1 (22.7–31.2)	26.6 (21.7–31.8)	25.6 (23.9–30.2)	1.000
Fat-free mass, kg	43.5 (41.6–47.6)	42.2 (40.6–46.7)	44.2 (42.8–49.4)	0.155
Phase angle, °	4.8 ± 0.6	4.8 ± 0.6	4.9 ± 0.7	0.676
Lymphedema-related measurements				
Right affected limb, *n* (%)	23 (47.9%)	12 (42.9%)	11 (55.0%)	0.559
Affected limb volume, mL	5088.5 (4375.2–5856.2)	4955.5 (4285.8–5763.0)	5181.0 (4446.2–6100.8)	0.385
Absolute interlimb volume difference, mL	579.5 (414.5–1202.0)	653.5 (454.8–1202.0)	507.5 (404.2–1160.0)	0.714
Relative interlimb volume difference, %	14.1 (9.6–26.3)	14.8 (10.2–26.3)	14.1 (9.3–25.6)	0.668
Dietary characteristics				
Mediterranean diet adherence score (PREDIMED-17)	8.7 ± 2.3	8.9 ± 2.0	8.3 ± 2.7	0.388
Energy intake, kcal/day	1881.6 ± 360.3	1885.1 ± 395.1	1876.5 ± 312.4	0.934
Protein intake, g/day	80.1 (63.4–93.6)	70.4 (61.2–98.6)	81.2 (71.8–86.7)	0.626
Fiber intake, g/day	14.9 (10.9–21.0)	13.6 (11.0–18.0)	16.8 (12.1–21.5)	0.374
Biochemical characteristics				
Fasting glucose, mg/dL	94.0 (89.0–101.0)	95.0 (89.0–104.0)	93.0 (89.0–97.0)	0.986
Total cholesterol, mg/dL	204.0 (180.2–228.0)	209.5 (182.0–228.8)	199.0 (173.0–222.0)	0.273
Triglycerides, mg/dL	105.0 (89.5–122.8)	102.0 (88.0–125.0)	107.5 (90.5–119.5)	0.921

Data are presented as mean ± SD or median (IQR), according to data distribution, and as *n* (%) for categorical variables. Between-group comparisons were performed using Welch’s *t*-test or the Mann–Whitney *U* test for continuous variables and Pearson’s chi-squared (χ^2^) test or Fisher’s exact test for categorical variables, as appropriate. Percentages were calculated based on available data for each variable. Statistical significance was set at *p* < 0.05. Significant *p*-values are shown in bold. BMI, body mass index; IQR, interquartile range; MET, metabolic equivalent of task; PREDIMED-17, 17-item PREDIMED-Plus questionnaire; SD, standard deviation.

**Table 2:** Changes from baseline in clinical, anthropometric, body composition, lymphedema and dietary outcomes at 3 and 6 months according to treatment group.

Outcome	Intervention Δ0–3 Months	Control Δ0–3 Months	Between-Group *p*-Value (3 Months)	Intervention Δ0–6 Months	Control Δ0–6 Months	Between-Group *p*-Value (6 Months)	Group × Time *p*-Value
Anthropometry and body composition							
Body weight, kg	−2.33 ± 1.99	−0.90 ± 2.08	**0.022**	−3.60 ± 2.98	−0.93 ± 2.44	**0.002**	**<0.001**
BMI, kg/m^2^	−0.86 ± 0.87	−0.36 ± 0.79	**0.044**	−1.46 ± 1.23	−0.39 ± 0.96	**0.002**	**0.001**
Waist circumference, cm	−2.00 (−4.25, −1.00)	−2.00 (−3.00, −0.75)	0.480	−3.00 (−5.88, −1.25)	−2.00 (−3.25, −0.75)	0.129	0.381
Fat mass, kg	−2.20 ± 2.04	−0.39 ± 2.01	**0.004**	−2.57 ± 2.73	−0.46 ± 2.41	**0.008**	**0.002**
Fat-free mass, kg	−0.55 ± 1.55	−0.45 ± 1.29	0.807	−0.57 ± 1.39	−0.01 ± 2.05	0.299	0.420
Phase angle, °	0.00 (−0.17, 0.18)	0.20 (−0.10, 0.30)	0.248	−0.05 (−0.30, 0.23)	−0.10 (−0.20, 0.20)	0.949	0.811
Lymphedema-related outcomes							
Affected limb volume, mL	−391.00 (−625.75, −64.25)	−248.50 (−416.50, 7.75)	0.503	−530.00 (−848.50, −303.50)	−407.50 (−682.25, −156.50)	0.540	0.714
Absolute interlimb volume difference, mL	−102.50 (−281.75, 180.50)	−214.50 (−339.25, −43.75)	0.352	−190.00 (−386.50, 38.50)	−255.50 (−450.25, −37.50)	0.561	0.708
Relative interlimb volume difference, %	−0.85 ± 10.47	−3.46 ± 11.06	0.414	−2.18 ± 11.63	−6.17 ± 8.97	0.191	0.388
Dietary outcomes							
Mediterranean diet adherence score (PREDIMED-17)	2.11 ± 3.19	3.00 ± 3.28	0.352	1.14 ± 4.18	3.10 ± 3.11	0.069	0.133
Energy intake, kcal/day	−249.26 ± 461.11	25.35 ± 431.92	**0.045**	−186.73 ± 400.65	14.52 ± 342.84	0.080	**0.040**

Data are presented as mean change ± SD or median change (IQR), according to data distribution. Changes from baseline at 3 and 6 months were calculated for each participant as the follow-up value minus the baseline value. Between-group differences in changes at each follow-up were assessed using Welch’s *t*-test or the Mann–Whitney U test, as appropriate. Longitudinal changes were analyzed using linear mixed-effects models including group, time, and the group × time interaction as fixed effects and participant as a random intercept. Analyses were based on available data for each outcome and time point. The *p*-value for the group × time interaction is reported in the last column. Statistical significance was set at a two-sided *p* < 0.05. Significant *p*-values are shown in bold. Δ0–3, change from baseline to 3 months; Δ0–6, change from baseline to 6 months; BMI, body mass index; IQR, interquartile range; PREDIMED-17, 17-item PREDIMED-Plus questionnaire; SD, standard deviation.

## Data Availability

The individual de-identified participant data that support the findings of this study are not publicly available. However, certain datasets may be made available upon reasonable request to the corresponding authors (S.G.-S. and M.I.Q.-O.), subject to institutional and ethical approvals. The data available for sharing include demographic, clinical, and outcome variables collected during the trial.

## Supplementary Materials

The following supporting information can be downloaded at: https://www.mdpi.com/article/10.3390/ijms27209008/s1.

## Abbreviations

The following abbreviations are used in this manuscript:

ASV	Amplicon sequence variant
BCRL	Breast cancer-related lymphedema
BIA	Bioelectrical impedance analysis
BMI	Body mass index
BPL1	*Bifidobacterium animalis* subsp. *lactis* CECT 8145
CFU	Colony-forming unit
FDR	False discovery rate
HOMA-IR	Homeostatic Model Assessment for Insulin Resistance
IPAQ	International Physical Activity Questionnaire
IQR	Interquartile range
MET	Metabolic equivalent of task
PCoA	Principal coordinates analysis
PCR	Polymerase chain reaction
PERMANOVA	Permutational multivariate analysis of variance
PERMDISP	Permutational analysis of multivariate dispersion
QIIME 2	Quantitative Insights Into Microbial Ecology 2
rRNA	Ribosomal RNA
SD	Standard deviation
SSPA	Public Health System of Andalusia
