# Supplementary material for: Longitudinal Gut Microbiota Changes in Women with Breast Cancer-Related Lymphedema Undergoing a Lifestyle Intervention
Source: Int J Mol Sci. 2026 Oct 11;27(20):9008. doi: 10.3390/ijms27209008 (PMC13705042; doi:10.3390/ijms27209008)
Supplement: Supplementary file 1 [file ijms-27-09008-s001.zip › Supplementary material .pdf]

Supplementary Material

Longitudinal Gut Microbiota Changes in Women with Breast Cancer-Related Lymphedema Undergoing a Lifestyle Intervention

Yolanda Ruiz-Molina [1,2,†], Marina Padial [3,4,†], Cristina Rodríguez-Díaz [4,5], Sheila González-Salvatierra [3,4,6,*], María del Mar Martín-Bravo [4,7], Isaac Plaza-Andrades [4,8,9], Lucía Aránega-Martín [4,8,9], Francisca Rodríguez-Pacheco [3,4,10], Alejandro Chicharro-Parras [7], Corina Verónica Sasso [2,3,4], María Isabel Queipo-Ortuño [4,8,9,*] and Gabriel Olveira [2,3,4,10]

[1] Servicio de Medicina Física y Rehabilitación, Hospital Universitario Virgen de la Victoria, 29010 Malaga, Spain; yorumoce@hotmail.com
[2] Department of Medicine and Dermatology, University of Málaga, 29010 Malaga, Spain; verosasso86@gmail.com (C.V.S.); gabrielm.olveira.sspa@juntadeandalucia.es (G.O.)
[3] Servicio de Endocrinología y Nutrición, Hospital Regional Universitario de Málaga, 29010 Malaga, Spain; mpadial27@gmail.com (M.P.); paqui.endocrino@gmail.com (F.R.-P.)
[4] IBIMA Plataforma BIONAND, Instituto de Investigación Biomédica de Málaga, 29010 Malaga, Spain; cris.rdrz@gmail.com (C.R.-D.); madelman77@hotmail.com (M.d.M.M.-B.); isaacplazaandrade@gmail.com (I.P.-A.); lucia.aramar97@uma.es (L.A.-M.)
[5] Unidad de Gestión Clínica de Aparato Digestivo, Hospital Universitario Virgen de la Victoria, 29010 Malaga, Spain
[6] Instituto de Salud Carlos III, CIBER of Frailty and Healthy Aging (CIBERFES), 28029 Madrid, Spain
[7] Servicio de Medicina Física y Rehabilitación, Hospital Regional Universitario de Málaga, 29010 Malaga, Spain; alejandrochicharroparras@gmail.com
[8] Unidad de Gestión Clínica de Oncología Médica, Hospital Universitario Virgen de la Victoria, 29010 Malaga, Spain
[9] Department of Surgical Specialties, Biochemistry and Immunology, University of Málaga, 29010 Malaga, Spain
[10] Instituto de Salud Carlos III, CIBER of Diabetes and Associated Metabolic Diseases (CIBERDEM), 28029 Madrid, Spain
* Correspondence: sheila.salvatierra@ibima.eu (S.G.-S.); iqueipo@uma.es (M.I.Q.-O.)
† These authors contributed equally to this work.

Supplementary Figures

Figure S1. Relative abundance of the 10 most abundant bacterial taxa in the intervention and control groups across follow-up.

Figure S2. Time-stratified differential abundance of selected bacterial taxa between intervention and control groups.

Figure S3. Relative abundance of the eight most abundant bacterial taxa according to supplementation status across follow-up.

Figure S4. *Bifidobacterium* abundance according to supplementation status across follow-up.

Figure S5. Bray–Curtis beta diversity according to supplementation adherence.

Figure S6. Bray–Curtis beta diversity according to weight-loss response.

Supplementary Tables

Table S1. Longitudinal and time-specific comparisons of alpha-diversity indices between the intervention and control groups.

Table S2. Exploratory differential abundance analysis of *Bifidobacterium* taxonomic assignments according to supplementation status.

Table S3. FDR-significant taxonomic differential abundance findings and distributional characteristics across exploratory analyses.

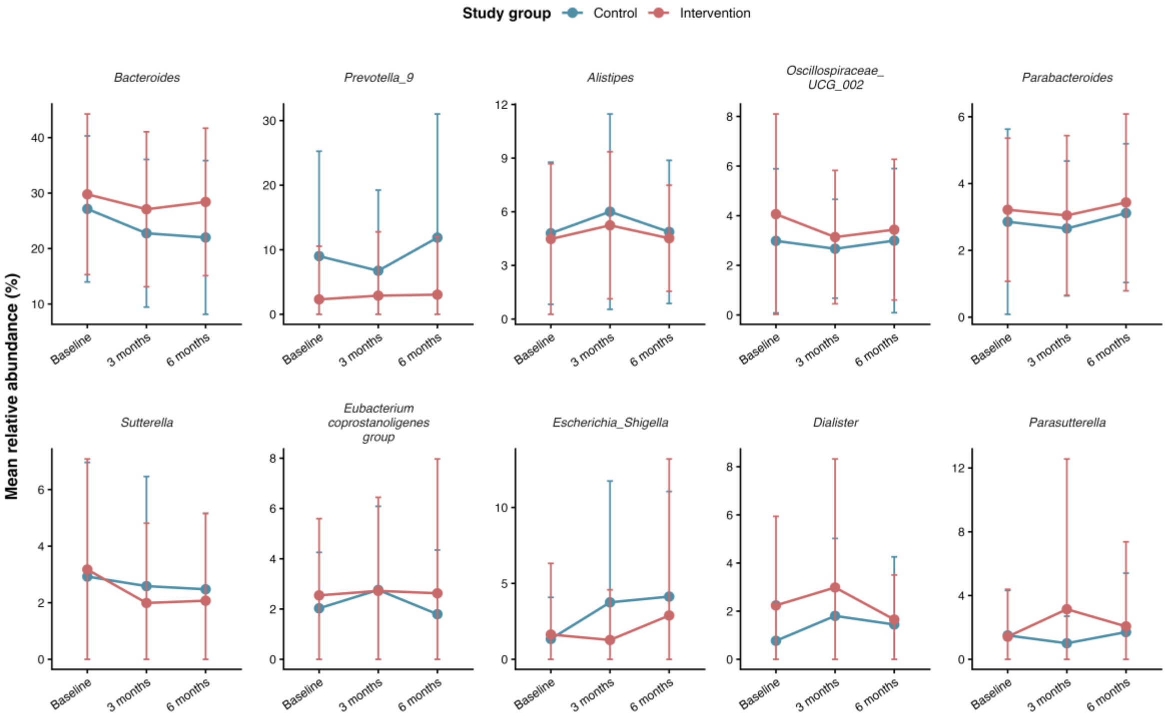

Figure S1. Relative abundance of the 10 most abundant bacterial taxa in the intervention and control groups across follow-up. Mean relative abundance (%) of the 10 most abundant taxa at baseline, 3 months, and 6 months according to study group. Error bars represent the standard deviation (SD).

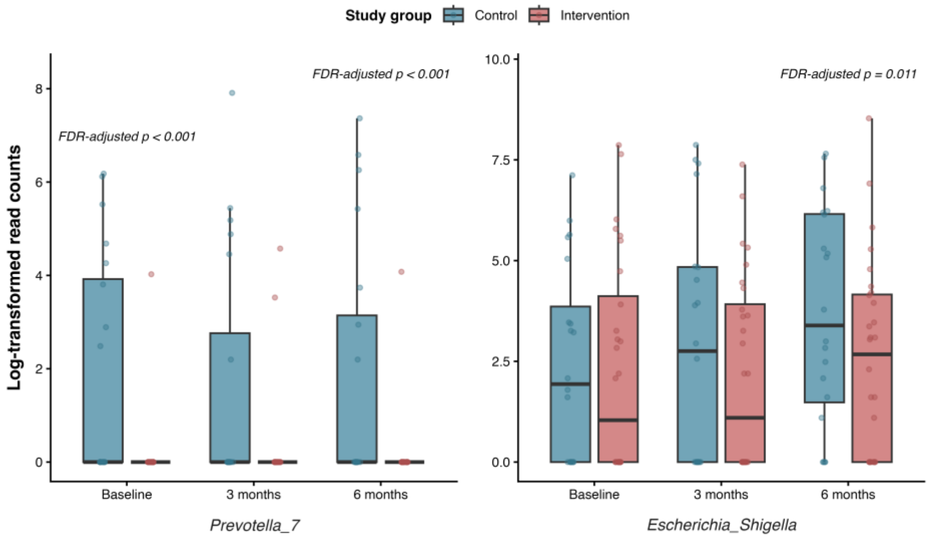

Figure S2. Time-stratified differential abundance of selected bacterial taxa between intervention and control groups. Boxplots show log-transformed read counts for taxa identified as significantly different between intervention and control groups in time-stratified DESeq2 analyses. *Prevotella_7* differed between groups at baseline and 6 months, whereas *Escherichia-Shigella* differed only at 6 months. No taxa remained significantly different at 3 months after FDR correction. Points represent individual samples. FDR-adjusted *p*-values are shown for significant comparisons.

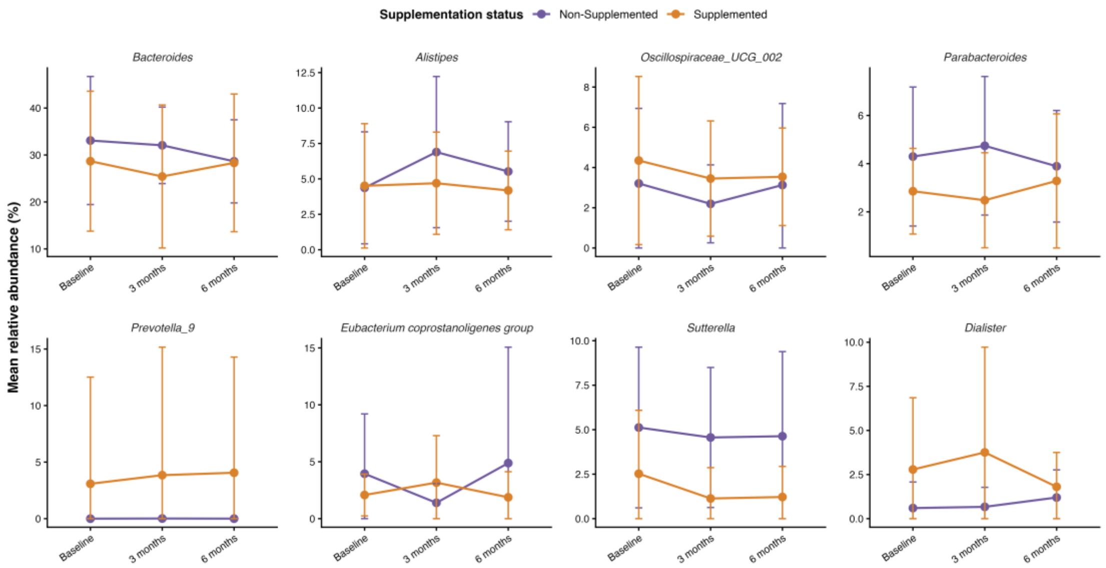

Figure S3. Relative abundance of the eight most abundant bacterial taxa according to supplementation status across follow-up. Mean relative abundance (%) of the eight most abundant bacterial taxa at baseline, 3 months, and 6 months among supplemented and non-supplemented participants within the intervention group. Points represent mean relative abundance, and error bars represent the standard deviation (SD).

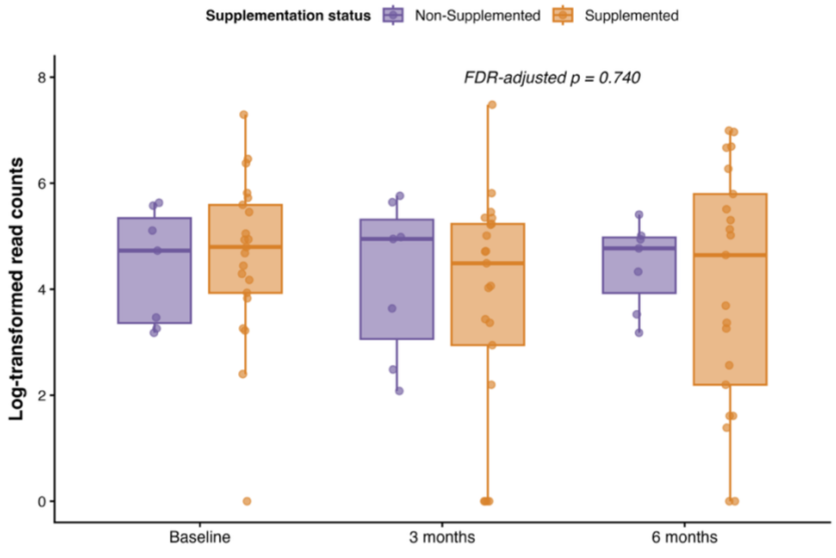

Figure S4. *Bifidobacterium* abundance according to supplementation status across follow-up. Boxplots show the distribution of log-transformed *Bifidobacterium* read counts at baseline, 3 months, and 6 months among supplemented and non-supplemented participants within the intervention group. Boxes represent the interquartile range, horizontal lines indicate the median, and points represent individual samples. In the DESeq2 analysis adjusted for time, *Bifidobacterium* abundance did not differ significantly according to supplementation status (FDR-adjusted $p = 0.740$). Given the non-viable nature of the administered BPL1 preparation, this analysis was exploratory and was not intended as a positive control or as an assessment of strain engraftment.

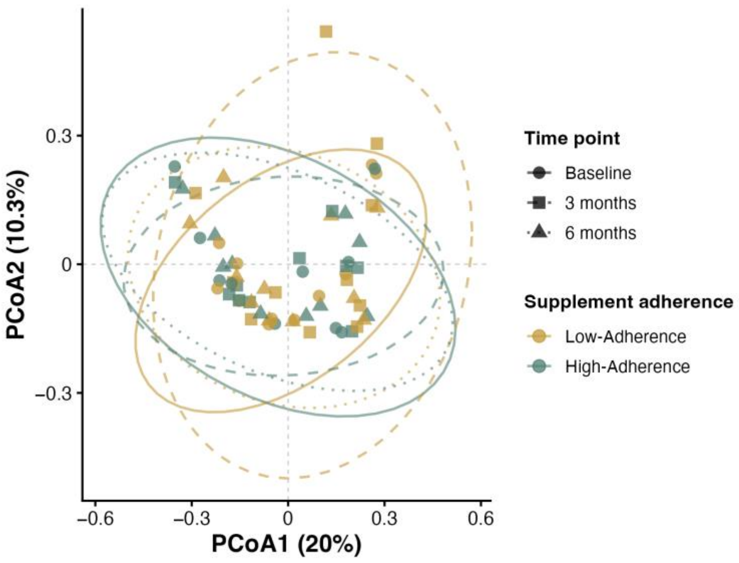

Figure S5. Bray–Curtis beta diversity according to supplementation adherence. Principal coordinate analysis (PCoA) based on Bray–Curtis dissimilarity showing gut microbial community composition in high- and low-adherence participants across follow-up. Points represent individual samples, with colors indicating adherence group and shapes indicating time points; 95% confidence ellipses are shown for each adherence group–time combination. PERMANOVA with permutations restricted by participant showed no significant adherence group × time effect on microbial community composition (R^2 = 0.012, F = 0.377, p = 0.537). Multivariate dispersion did not differ significantly among adherence group–time combinations (PERMDISP, p = 0.792).

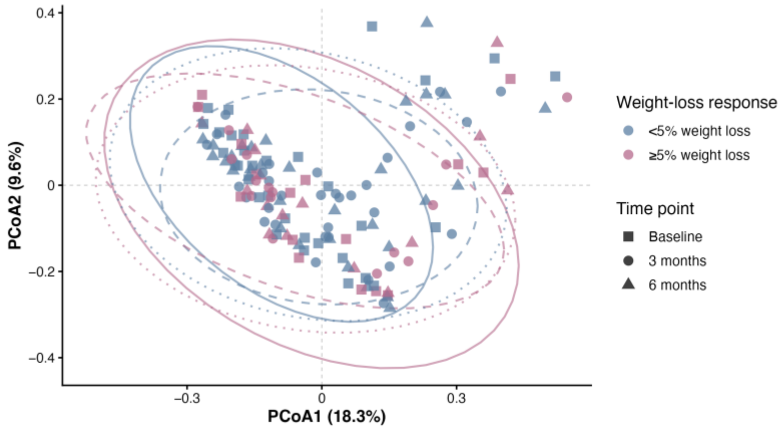

Figure S6. Bray–Curtis beta diversity according to weight-loss response. Principal coordinate analysis (PCoA) based on Bray–Curtis dissimilarity showing gut microbial community composition according to weight-loss response across baseline, 3 months, and 6 months. Points represent individual samples, colors indicate weight-loss response, and shapes indicate time point. PERMANOVA with permutations restricted by participant showed no significant weight-loss response × time interaction (R^2 = 0.007, F = 0.496, p = 0.099). Multivariate dispersion did not differ significantly among weight-loss response–time combinations (PERMDISP, p = 0.491).

Table S1. Longitudinal and time-specific comparisons of alpha-diversity indices between the intervention and control groups.

Alpha diversity index	Group ×Time (*p*-Value)	Holm-adjusted (*p*-Value)	Baseline (*p*-Value)	3 months (*p*-Value)	6 months (*p*-Value)	Δ0–3 months (*p*-Value)	Δ0–6 months (*p*-Value)
Chao1 richness	0.562	1.000	0.305	0.746	0.363	0.427	0.950
Shannon diversity index	0.430	1.000	0.198	0.975	0.699	0.445	0.810
Simpson index	0.563	1.000	0.421	0.826	0.826	0.683	0.859
Inverse Simpson diversity index	0.679	1.000	0.421	0.826	0.826	0.714	0.714

Alpha-diversity indices were calculated from count data aggregated at the genus level or, when genus-level assignment was unavailable, at the nearest resolved taxonomic level, after rarefaction to an even sequencing depth of 3000 reads per sample. Longitudinal changes were evaluated using linear mixed-effects models including group, time, and the group × time interaction as fixed effects and participant as a random intercept. The overall group × time interaction was assessed using a likelihood-ratio test comparing models with and without the interaction term, and corresponding *p*-values were adjusted across the four alpha-diversity indices using the Holm method. Baseline, 3-month, and 6-month comparisons were based on alpha-diversity values at each respective time point and were assessed using the Mann–Whitney *U* test. Δ0–3 and Δ0–6 represent changes from baseline to 3 and 6 months, respectively, calculated for each participant and compared between groups using the Mann–Whitney *U* test. All tests were two-sided, and statistical significance was set at $p < 0.05$. Δ, change from baseline.

Table S2. Exploratory differential abundance analysis of *Bifidobacterium* taxonomic assignments according to supplementation status.

**Taxonomic assignment**	**Positive samples, supplemented, *n*/*N* (%)**	**Positive samples, non-supplemented, *n*/*N* (%)**	**Mean normalized count, supplemented**	**Mean normalized count, non-supplemented**	**Base mean**	**Unshrunken log2FC**	***p*-Value**	**FDR**
Bifidobacterium adolescentis	45/63 (71.4%)	13/21 (61.9%)	135.83	64.79	118.07	1.013	0.171	0.597
Bifidobacterium bifidum	22/63 (34.9%)	1/21 (4.8%)	11.22	2.25	8.43	5.698	<0.001	<0.001
Bifidobacterium breve	19/63 (30.2%)	11/21 (52.4%)	26.10	55.58	33.47	−1.089	0.406	0.794
Bifidobacterium catenulatum	6/63 (9.5%)	5/21 (23.8%)	5.44	14.87	7.80	−1.772	0.481	0.794
Bifidobacterium dentium	2/63 (3.2%)	2/21 (9.5%)	0.62	1.96	0.35	−1.923	0.567	0.794
Bifidobacterium animalis	3/63 (4.8%)	1/21 (4.8%)	0.52	0.30	0.31	0.021	0.995	1.000

Positive-sample frequencies and group-specific mean normalized counts were calculated from the observed counts before DESeq2 outlier replacement. The base mean and differential abundance estimates correspond to the fitted DESeq2 model, including its standard automatic outlier replacement procedure. The log2FC values presented here are unshrunken DESeq2 estimates; the corresponding shrunken estimate for the FDR-significant *Bifidobacterium bifidum* association is provided in Table S3A. Positive log2FC values indicate higher abundance in supplemented participants, whereas negative values indicate higher abundance in non-supplemented participants. *p*-values were adjusted using the Benjamini–Hochberg false discovery rate procedure. Eight *Bifidobacterium* taxonomic assignments were available for the exploratory analysis. Seven assignments had valid *p*-values and were included in the Benjamini–Hochberg multiple-testing correction. Six species-level assignments are presented in the table; one additional assignment resolved only at the genus level (*Bifidobacterium*) was included in the multiple-testing correction but is not shown because it could not be assigned to species level. *Bifidobacterium pullorum* yielded no estimable adjusted *p*-value and was therefore not included in the multiple-testing correction. FDR, false discovery rate; log2FC, log2 fold change; *n*, number of positive samples; *N*, total number of samples in the corresponding group.

Table S3. FDR-significant taxonomic differential abundance findings and distributional characteristics across exploratory analyses.

Table S3A. FDR-significant taxonomic differential abundance findings and log2 fold-change shrinkage across exploratory analyses.

Analysis	Time/Contrast	Taxon	Rank	Direction	Base mean	Unshrunken log2FC	Shrunken log2FC	SE	*p*-Value	FDR
Intervention *vs* Control	Overall	Barnesiellaceae	Family	Higher Intervention	16.48	3.132	2.248	0.639	9.68×10^{-7}	5.76×10^{-5}
Intervention *vs* Control	Overall	Desulfovibrionaceae	Family	Higher Intervention	11.04	1.837	1.443	0.537	6.28×10^{-4}	0.019
Intervention *vs* Control	Overall	*Lachnospiraceae_UCG_010*	Genus-level group	Higher Control	28.97	−1.811	−1.419	0.507	3.52×10^{-4}	0.014
Intervention *vs* Control	Overall	*Prevotella_7*	Genus	Higher Control	20.82	−11.147	−1.946	1.316	2.50×10^{-17}	2.98×10^{-15}
Intervention *vs* Control	BL→M3 group × time	Izemoplasmatales	Order	Greater BL→M3 increase in Control[1]	45.77	−11.448	0.042	2.173	1.38×10^{-7}	1.65×10^{-5}
Intervention *vs* Control	BL→M3 group × time	*Frisingicoccus*	Genus	Greater BL→M3 increase in Control[1]	3.21	−24.419	−0.127	7.206	7.03×10^{-4}	0.042
Intervention *vs* Control	Baseline	*Prevotella_7*	Genus	Higher Control	26.66	−25.355	−2.516	1.774	2.45×10^{-46}	2.82×10^{-44}
Intervention *vs* Control	6 months	*Prevotella_7*	Genus	Higher Control	22.72	−25.456	−2.079	2.190	3.05×10^{-31}	3.97×10^{-29}
Intervention *vs* Control	6 months	*Escherichia-Shigella*	Genus-level group	Higher Control	196.71	−3.525	−0.169	0.935	1.63×10^{-4}	0.011
Supplementation	Overall	*Klebsiella*	Genus	Higher Supplemented	58.44	7.491	0.932	1.851	5.18×10^{-5}	0.003
Supplementation	Overall	*Prevotella_9*	Genus	Higher Supplemented	82.14	8.086	3.626	1.479	4.57×10^{-8}	5.49×10^{-6}

Analysis	Time/Contrast	Taxon	Rank	Direction	Base mean	Unshrunken log2FC	Shrunken log2FC	SE	*p*-Value	FDR
Supplementation	Baseline	*Klebsiella*	Genus	Higher Supplemented	26.69	22.055	1.445	2.659	1.08×10^{-16}	6.65×10^{-15}
Supplementation	Baseline	*Prevotella_9*	Genus	Higher Supplemented	39.92	22.616	1.795	2.414	7.31×10^{-21}	8.99×10^{-19}
Supplementation – species level	Overall	*Bifidobacterium bifidum*	Species-level assignment	Higher Supplemented	8.43	5.698	1.707	1.423	6.21×10^{-5}	4.35×10^{-4}
Adherence	Overall	*Rikenellaceae_RC9_gut_group*	Genus-level group	Higher High-Adherence	19.64	26.612	0.149	2.918	7.61×10^{-20}	9.06×10^{-18}
Adherence	Overall	*Escherichia-Shigella*	Genus-level group	Higher High-Adherence	329.82	4.379	2.521	0.996	1.10×10^{-5}	4.87×10^{-4}
Adherence	Overall	*Prevotella_9*	Genus	Higher Low-Adherence	446.81	−5.929	−2.080	1.356	1.23×10^{-5}	4.87×10^{-4}
Adherence	Overall	*Parasutterella*	Genus	Higher Low-Adherence	193.00	−2.901	−2.572	0.743	9.46×10^{-5}	0.003
Adherence	Overall	*Lachnospiraceae_AC2044_group*	Genus-level group	Higher Low-Adherence	6.37	−5.974	−3.235	1.748	6.31×10^{-4}	0.015
Adherence	6 months	*Clostridium_sensu_stricto_1*	Genus	Higher High-Adherence	11.52	6.967	0.445	1.790	9.89×10^{-5}	0.012

Differential abundance was assessed using DESeq2 on non-rarefied count data. For genus/nearest-resolved-level analyses, taxa present in at least 10% of samples within the corresponding analysis set were retained. Sampling time was included as a covariate in the overall analyses. For the main intervention-versus-control comparison, participant-blocked longitudinal models were additionally used to evaluate group × time interactions. For FDR-significant taxa, log2 fold-change estimates were stabilized using the DESeq2 *lfcShrink* function with the normal prior. Both unshrunken and shrunken log2FC estimates are presented for transparency. Statistical significance was determined from the original DESeq2 hypothesis tests; therefore, *p*-values and Benjamini–Hochberg FDR-adjusted *p*-values correspond to the original hypothesis tests and were not recalculated after log2FC shrinkage. The SE corresponds to the standard error of the original unshrunken DESeq2 coefficient. For cross-sectional contrasts, direction indicates the group with higher abundance. [1]For group × time interactions, direction refers to the original unshrunken interaction coefficient and describes the relative difference in change from baseline to 3 months between groups. Supplementation-status and supplement-adherence analyses were secondary

and hypothesis-generating because supplementation was optional and adherence was not randomized. BL, baseline; M3, 3 months; FDR, false discovery rate; log2FC, log2 fold change; SE, standard error.

Analysis-set summary. Intervention versus control: overall, 119 taxa tested/4 FDR-significant; baseline, 115/1; 3 months, 123/0; 6 months, 130/2; participant-blocked BL→M3, 119/2; participant-blocked BL→M6, 119/0. Supplementation: overall, 120/2; baseline, 124/2; 3 months, 121/0; 6 months, 130/0. Adherence: overall, 119/5; baseline, 110/0; 3 months, 116/0; 6 months, 119/1. Weight-loss response: overall, 119/0; baseline, 115/0; 3 months, 123/0; 6 months, 130/0. The exploratory *Bifidobacterium* analysis included seven taxonomic assignments with valid *p*-values in the multiple-testing correction; six species-level assignments are presented in Supplementary Table S2, of which one was FDR-significant.

Table S3B. Prevalence and zero-count frequencies of FDR-significant taxa across differential abundance analyses.

Analysis	Time/Contrast	Taxon	Group	Prevalence (non-zero samples), *n/N* (%)	Zero-count samples, *n/N* (%)
Intervention *vs* Control	Overall	Barnesiellaceae	Intervention	50/84 (59.5%)	34/84 (40.5%)
			Control	13/60 (21.7%)	47/60 (78.3%)
Intervention *vs* Control	Overall	Desulfovibrionaceae	Intervention	54/84 (64.3%)	30/84 (35.7%)
			Control	19/60 (31.7%)	41/60 (68.3%)
Intervention *vs* Control	Overall	*Lachnospiraceae_UCG_010*	Intervention	51/84 (60.7%)	33/84 (39.3%)
			Control	39/60 (65.0%)	21/60 (35.0%)
Intervention *vs* Control	Overall	*Prevotella_7*	Intervention	4/84 (4.8%)	80/84 (95.2%)
			Control	21/60 (35.0%)	39/60 (65.0%)
Intervention *vs* Control	Baseline	*Prevotella_7*	Intervention	1/28 (3.6%)	27/28 (96.4%)
			Control	8/20 (40.0%)	12/20 (60.0%)
Intervention *vs* Control	6 months	*Prevotella_7*	Intervention	1/28 (3.6%)	27/28 (96.4%)
			Control	7/20 (35.0%)	13/20 (65.0%)
Intervention *vs* Control	6 months	*Escherichia-Shigella*	Intervention	18/28 (64.3%)	10/28 (35.7%)
			Control	16/20 (80.0%)	4/20 (20.0%)

Analysis	Time/Contrast	Taxon	Group	Prevalence (non-zero samples), *n*/*N* (%)	Zero-count samples, *n*/*N* (%)
Intervention *vs* Control	BL→M3 interaction	Izemoplasmatales	Intervention – BL	12/28 (42.9%)	16/28 (57.1%)
			Intervention – M3	13/28 (46.4%)	15/28 (53.6%)
			Control – BL	3/20 (15.0%)	17/20 (85.0%)
			Control – M3	7/20 (35.0%)	13/20 (65.0%)
Intervention *vs* Control	BL→M3 interaction	*Frisingicoccus*	Intervention – BL	6/28 (21.4%)	22/28 (78.6%)
			Intervention – M3	2/28 (7.1%)	26/28 (92.9%)
			Control – BL	1/20 (5.0%)	19/20 (95.0%)
			Control – M3	7/20 (35.0%)	13/20 (65.0%)
Supplementation status	Overall	*Klebsiella*	Supplemented	19/63 (30.2%)	44/63 (69.8%)
			Non-supplemented	2/21 (9.5%)	19/21 (90.5%)
Supplementation status	Overall	*Prevotella_9*	Supplemented	26/63 (41.3%)	37/63 (58.7%)
			Non-supplemented	2/21 (9.5%)	19/21 (90.5%)
Supplementation status	Baseline	*Klebsiella*	Supplemented	7/21 (33.3%)	14/21 (66.7%)
			Non-supplemented	0/7 (0.0%)	7/7 (100.0%)
Supplementation status	Baseline	*Prevotella_9*	Supplemented	9/21 (42.9%)	12/21 (57.1%)
			Non-supplemented	0/7 (0.0%)	7/7 (100.0%)
Supplementation status – species level	Overall	*Bifidobacterium bifidum*	Supplemented	22/63 (34.9%)	41/63 (65.1%)
			Non-supplemented	1/21 (4.8%)	20/21 (95.2%)
Supplement adherence	Overall	*Rikenellaceae_RC9_gut_group*	High adherence	6/30 (20.0%)	24/30 (80.0%)
			Low adherence	3/33 (9.1%)	30/33 (90.9%)

Analysis	Time/Contrast	Taxon	Group	Prevalence (non-zero samples), *n*/*N* (%)	Zero-count samples, *n*/*N* (%)
Supplement adherence	Overall	*Escherichia-Shigella*	High adherence	20/30 (66.7%)	10/30 (33.3%)
			Low adherence	17/33 (51.5%)	16/33 (48.5%)
Supplement adherence	Overall	*Prevotella_9*	High adherence	9/30 (30.0%)	21/30 (70.0%)
			Low adherence	17/33 (51.5%)	16/33 (48.5%)
Supplement adherence	Overall	*Parasutterella*	High adherence	18/30 (60.0%)	12/30 (40.0%)
			Low adherence	27/33 (81.8%)	6/33 (18.2%)
Supplement adherence	Overall	*Lachnospiraceae_AC2044_group*	High adherence	0/30 (0.0%)	30/30 (100.0%)
			Low adherence	10/33 (30.3%)	23/33 (69.7%)
Supplement adherence	6 months	*Clostridium_sensu_stricto_1*	High adherence	6/10 (60.0%)	4/10 (40.0%)
			Low adherence	1/11 (9.1%)	10/11 (90.9%)

Prevalence was defined as the number and percentage of samples with non-zero counts, whereas zero-count frequency represents the number and percentage of samples with zero counts within the corresponding analytical group and time point. For overall analyses, prevalence and zero-count frequencies were calculated across all available longitudinal samples within each comparison group. For FDR-significant group × time interactions, distributions are shown separately for the time points involved in the corresponding interaction. These descriptive measures are provided to contextualize differential abundance findings in relation to taxon sparsity and do not represent parameters estimated from a formal zero-inflated model. BL, baseline; FDR, false discovery rate; M3, 3 months; M6, 6 months; *n*, number of samples with the indicated count status; *N*, total number of samples in the corresponding group.
